# Morphology Transition Engineering of ZnO Nanorods to Nanoplatelets Grafted Mo_8_O_23_-MoO_2_ by Polyoxometalates: Mechanism and Possible Applicability to other Oxides

**DOI:** 10.1038/s41598-017-05750-x

**Published:** 2017-07-19

**Authors:** Ahmed H. Abdelmohsen, Waleed M. A. El Rouby, Nahla Ismail, Ahmed A. Farghali

**Affiliations:** 1 Materials Science and Nanotechnology Department, Faculty of Postgraduate Studies for Advanced Science (PSAS), Beni-Suef University, 62511 Beni-Suef, Egypt; 2 Augsburg University, Institute of Physics, Universitätsstrass 1, 86159 Augsburg, Germany; 3 Institute of Condensed Matter and Nanosciences (IMCN), Bio- and Soft Matter, Université Catholique de Louvain, Louvain la Neuve, B-1348 Belgium; 4 Physical Chemistry Department, Centre of Excellence for Advanced Sciences, Renewable Energy Group, National Research Centre, 12311 Dokki, Giza Egypt

**Keywords:** Two-dimensional materials, Molecular self-assembly

## Abstract

A new fundamental mechanism for reliable engineering of zinc oxide (ZnO) nanorods to nanoplatelets grafted Mo_8_O_23_-MoO_2_ mixed oxide with controlled morphology, composition and precise understanding of the nanoscale reaction mechanism was developed. These hybrid nanomaterials are gaining interest due to their potential use for energy, catalysis, biomedical and other applications. As an introductory section, we demonstrate a new expansion for the concept ‘materials engineering’ by discussing the fabrication of metal oxides nanostructures by bottom-up approach and carbon nanoparticles by top-down approach. Moreover, we propose a detailed mechanism for the novel phenomenon that was experienced by ZnO nanorods when treated with phosphomolybdic acid (PMA) under ultra-sonication stimulus. This approach is expected to be the basis of a competitive fabrication approach to 2D hybrid nanostructures. We will also discuss a proposed mechanism for the catalytic deposition of Mo_8_O_23_-MoO_2_ mixed oxide over ZnO nanoplatelets. A series of selection rules (SRs) which applied to ZnO to experience morphology transition and constitute theory for morphology transition engineering (TMTE) will be demonstrated through the article, besides a brief discussion about possibility of other oxides to obey this theory.

## Introduction

**Figure 1:**
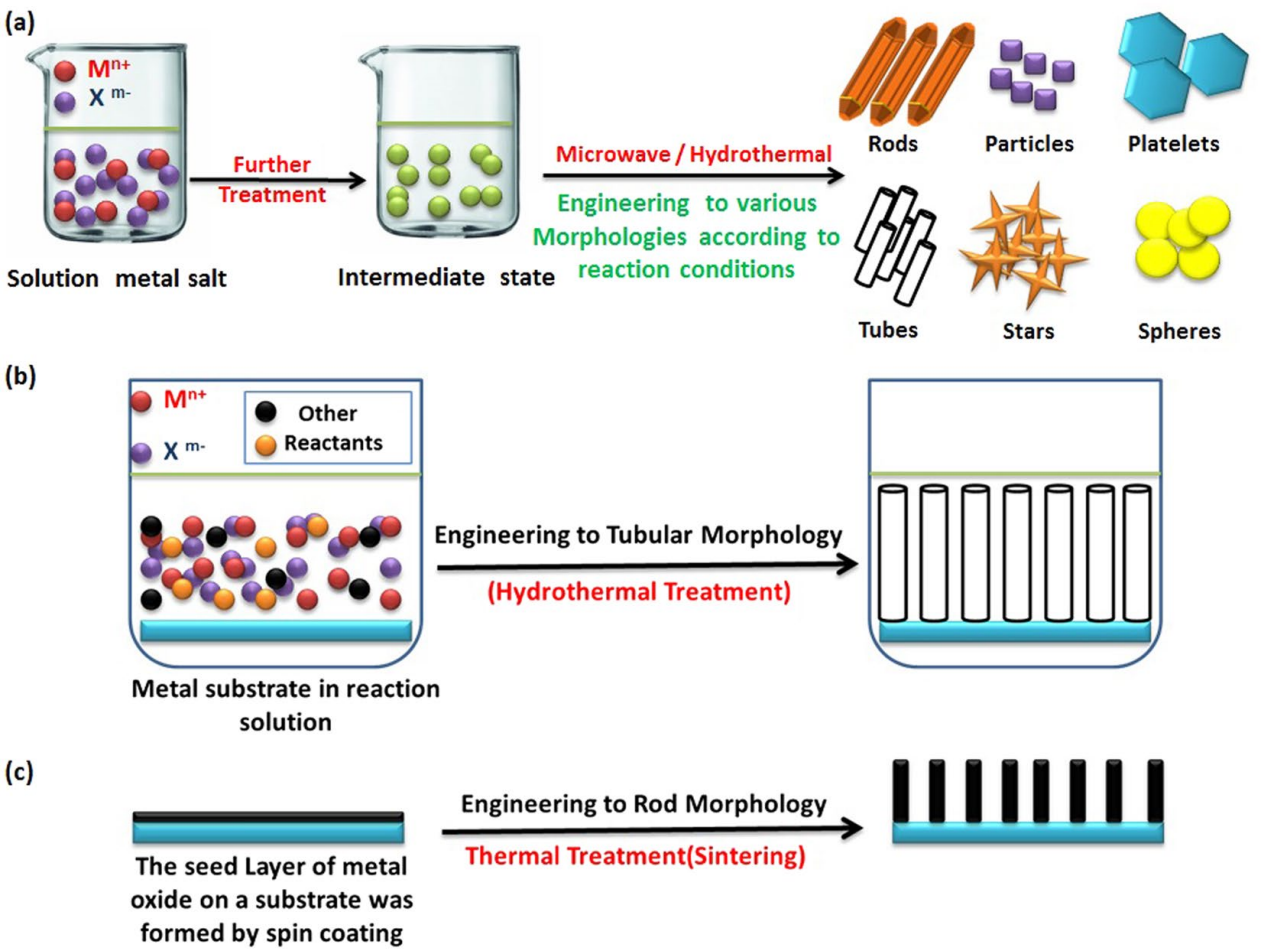
Schematic illustration for the morphology engineering of metal oxides by bottom-up approach.

Here, *Abdelmohsen et al*. expand the concept of ‘materials engineering’^1^ to include “controlling and designing the oriented structures of materials by re-scaling their dimensions^2–4^ or varying their external morphologies^5–7^, favorably with functionalization^8–10^, decoration^11–14^, doping^15–18^ or mixing^19, 20^ with other materials to attend the synergistic effect^21–23^ which enhance their properties”. Top-down and bottom-up approaches are the main two methods used in nanofabrication. The bottom-up approach has more advantages than the top-down approach because the former has a better chance of fabricating structures with homogenous chemical composition, less defects, and better short- and long-range ordering^24^. Depending on experimental results were observed by our group and other reported papers^25–43^, we demonstrate the following examples to explain top-down and bottom-up approaches for engineering of various nanomaterials. Firstly, Fig. 1 illustrates the engineering of metal oxides atom by atom in solution which gives rise to crystal planes, crystal planes further stack onto each other, resulting in various nanostructures. According to the reaction conditions and reactants, the starting precursors can be engineered to miscellaneous nanostructures like particles^2–4^, rods^5, 11–14^, tubes^27–29^, thin films^30–32^, plates^33–35^, stars^36–38^, wires^39–42^, belts^16, 42^ or hollow spheres^28, 43^. This method is known as bottom-up approach. We have used this approach to prepare different metal oxides nanostructures like (ZnO, CuO, SnO, Fe_2_O_3_, and Co_3_O_4_) and we have examined their behaviors when react with polyoxometalates (POMs) under ultra-sonication stimulus. This will be discussed at the end of article.

**Figure 2:**
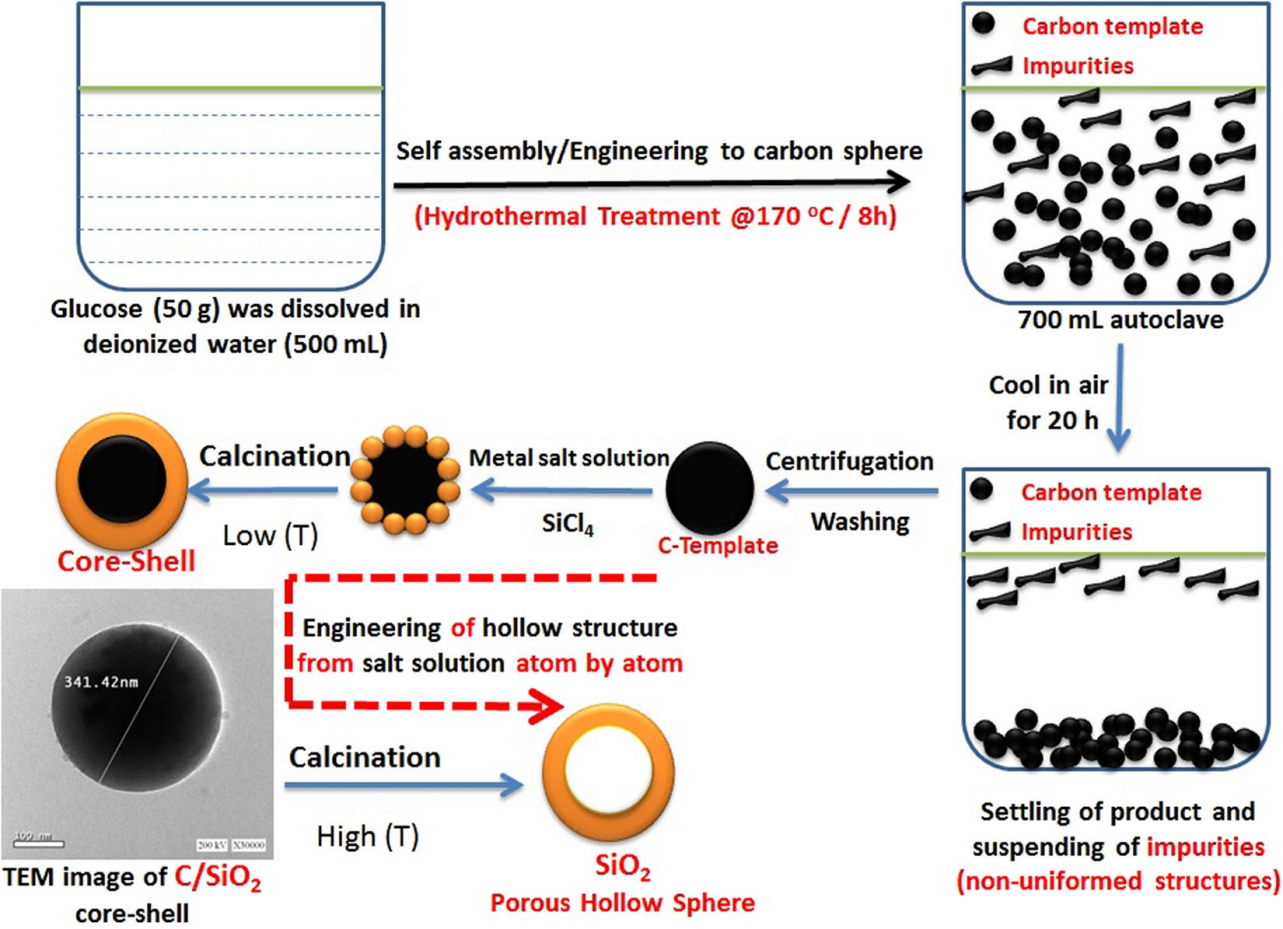
Schematic illustration for the morphology engineering of carbon template from sucrose solution, then using the template to engineer the core-shell and hollow structure from salt solution (bottom-up approach).

Additionally, we have used soft-template approach for engineering C/SiO_2_ core shell sphere. This approach is a widely used for synthesizing core-shell and hollow structures^43^. This bottom-up method involves the adsorption of metal cations on the surface of carbon template functionalized with hydroxyl groups. The carbon template acts as a skeleton to engineer the hollow structure of metal oxide. Further calcination removes the template and creates pores through the wall as shown in Fig. 2 (Supplementary - Figure S1).

**Figure 3:**
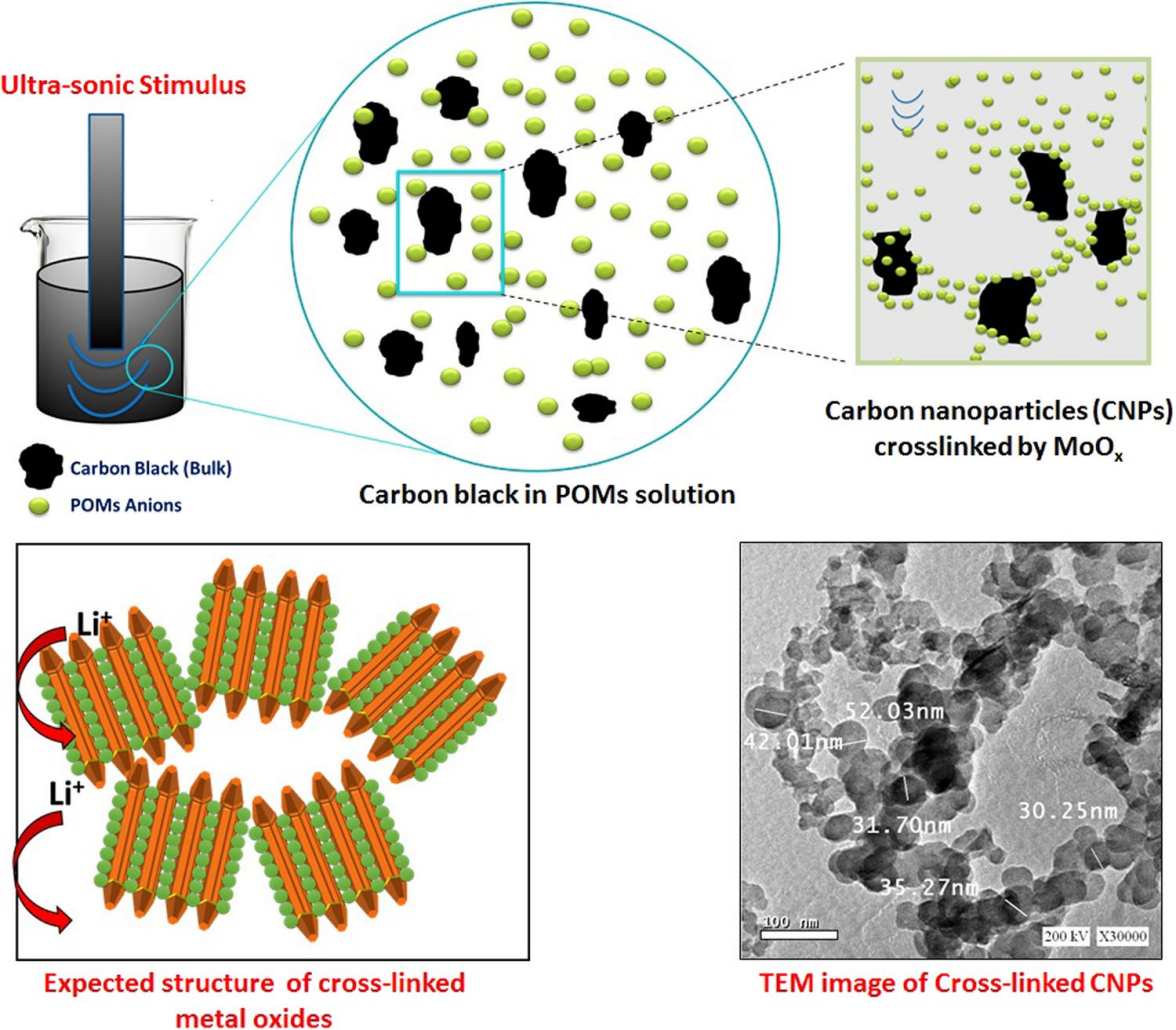
Schematic illustration for the morphology engineering of carbon nanoparticles (CNPs) by top-down approach [Polyoxometalate assisted-solution technique]. Expected structure before SEM inspection of real results is shown down-left.

Secondly, Fig. 3 explains the fabrication of carbon nanoparticles by top-down approach^44^ which involves gradual division (etching) of the initially large carbon black to smaller particles by ultra-sonication. The etched particles are stabilized by the strangely adsorbed negatively charged polyoxometalate (POM) monolayer that deposited as MoO_x_ forming cross-linked carbon nanoparticles^44^. Further treatment produces the colloidal carbon nanoparticles (CNPs) (Supplementary - Figure S2). By imitating this approach, *Abdelmohsen* has conceived the idea of crosslinked metal oxides to be used as anodes in lithium ion batteries that was achieved by using metal oxides as a precursor instead of carbon black at the same reaction conditions. Due to the differences in surface chemistry of metal oxides in comparing with that of carbon black, a further self-assembly of ZnO nanorods resulted in a morphology transition (MT) to ZnO nanoplatelets grafted Mo_8_O_23_-MoO_2_ mixed oxide (Supplementary Figure S3).

Controlled oriented morphology change is an important phenomenon in nanoscience and nanotechnology. Many physical and chemical properties of these nanomaterials are dictated by the size, shape, as well as the surface structure and chemistry. Thus morphology is a crucial parameter that controls the property and functionality of the materials^5, 45–49^. A number of approaches, including sol-gel synthesis^50^, template method^51^, thermal decomposition^52^, hydrothermal^53^, co-precipitation^54^, and electrodeposition^55^ have been proposed for the preparation of transition metal oxides with controlled morphology and surface chemistry. Yet, there are still many aspects to be unraveled regarding the nanoscale growth mechanism, solid-state surface-surface interactions and crystal lattice match that could finally lead to precise design oriented nanomaterials synthesis.

Amongst the main studied transition-metal oxides, zinc oxide (ZnO) and molybdenum oxides have attracted much attention for their potential application in lithium-ion batteries^56, 57^, supercapacitors^58^, dye removal^50, 51^, pigments^59^, gas sensors^60, 61^, and light emitting devices^62, 63^. In particular, ZnO has the potential for many applications due to its unique physical and chemical properties, such as high energy density, high electrochemical coupling coefficient, and broad range of radiation absorption^64^. In addition, it can be synthesized in a variety of nanoscale shapes including one- (1D), two- (2D), and three-dimensional (3D) structures^64^. Many approaches have been tested so far for the preparation of (2D) ZnO nanostructures such as vapor transport process^65, 66^, thermal evaporation^24^, carbothermal reduction process^67–69^, chemical bath deposition or hydro-thermal methods^70, 71^. Unfortunately, these approaches are based on complicated procedures, high temperature processes, and do not provide controllable and reproducible results. All important, nanoscale dimensions are rarely reached and thick objects (platelets) are obtained^33^. The ability to readily and reliably synthesize 2D ZnO nanostructures is thus required to further exploit the peculiar performances of this material. Additionally, the surface decoration of ZnO nanostructures is expected to further enhance the performance of the nanocomposites^11–14^. For example, the synthesis of (2D) ZnO nanoplatelets covered with molybdenum oxide is expected to boost up the physico-chemical properties of the composite due to the synergistic effect of the components^21, 22^.

Polyoxometalates (POMs) and their related compounds are considered as an attractive class of materials with many unique functions of catalysis due to their particular structural and electronic properties^72^. These have been also used for spontaneous self-assembled growth of micro and nanomaterials^73, 74^. For instance Das *et al*.^5^ studied the morphology evolution in hexagonal V_10_O_28_ - type polyoxometalate macrocrystals as a function of sonication temperatures. The morphology evolved from nano-rods to microflowers passing through intermediate hexagonal shaped microcrystals as the sonication temperature was raised from 50 to 80 °C. Among POMs, phosphomolybdic acid (PMA) is constructed by MoO_6_ octahedron and considered as good precursors to synthesize the mixed oxides with good homogeneity in the distribution^51^. Since, the nanoscale morphology of metal oxides alters the physical and chemical properties as compared to bulk materials, facile synthesis methods that can allow great control over the morphology and crystal architecture are of great interest.

Here in, a polyoxometalates-assisted solution technique is set forward as the basis for a competitive fabrication method of 2D metal oxides nanostructures. To our knowledge, similar process has not been reported yet, with only few studies related to engineering the morphology transition of ZnO nanostructures being reported so far^75–78^. The obtained composite is consisting of two main components; one is the decorating ZnO nanorods that may have flowers or cages like structures, while the second component is the (ZnO nanoplatelets grafted Mo_8_O_23_-MoO_2_ mixed oxide) that has good size distribution through the whole sample.

These nanocomposites are expected to have a great importance of fabricating functional nanodevices due to their high surface to volume ratio, nano scale thickness, as well as potentially interesting optical and photocatalytic properties^79^. They are expected to be used in different applications. For instance, ZnO Nanoplatelets plays a significant role in photocatalytic degradation of organic dyes especially ones with thin thickness. In our case, we expected also good performance due to the contribution of molybdenum oxides that have high catalytic activity^79^. Moreover, photocatalytic hydrogen production system using zinc oxide as a photocatalyst typically suffers from low efficiency because of the wide band gap of ZnO. Several studies reported that ZnO under visible light can be significantly enhanced by using doped ZnO nanomaterials. For instance, Yuan *et al*.^80^ found that the 0.5 wt% ZnTCPP-MoS_2_/ZnO photocatalyst shows the maximum H_2_ evolution rate of 75 µmol/h g, which is higher than that of 2.0 wt% ZnTCPP-Pt/ZnO photocatalyst, indicating that the layered MoS_2_ acts as more effective cocatalyst than the commonly used Pt nanoparticles. Hence, we also expect a competitive performance of our novel nanocomposite. From another prospect, this hybrid material is considered the next generation materials for lithium ion batteries. Pure ZnO nanoplatelets electrode exhibited good cyclability and delivered a reversible discharge capacity of 368 mAh/g after 100 cycles at 0.1 C^81^. Additionally, MoO_x_ has high capacity for different architecture^82–86^. Due to synergetic effect, we expect a high performance for the novel ZnO grafted Mo_8_O_23_-MoO_2_ mixed oxide nanocomposite. We also predict its application for data storage, memory devices and organic synthesis, optoelectronic fields which may improve the performance of these devices^87, 88^. Generally, the term ‘nanocomposite’^89^ can be defined as “A mixture of different materials, that are mixed by different ways (mechanically^90–92^, direct growth of one material over another one^93–95^, encapsulation of one inside other^96–98^, electrostatic attraction between two materials^99, 100^, deposition of material over another one^101–103^… etc.) inwhich the synergistic effect^104^ can be achieved to enhance the materials properties. Moreover, ‘the synergistic effect’^21–23^ can be defined as “An effect that is observed in composites, in which one property of certain component can reveal withdraw back of other component, or enhance the same property within the whole composite, which finally lead to a competitive performance in comparing with that of the individual components”. An example for revealing the disadvantages of components and enhancing the whole composite performance is represented in the role of carbonaceous materials when mixed with metal oxides to act as anodes for lithium ion batteries^98–100^. The carbonaceous materials reveal pulverization of metal oxides and contribute to the whole capacity and electrical conductivity of the composite.

## Results and Discussion

**Figure 4:**
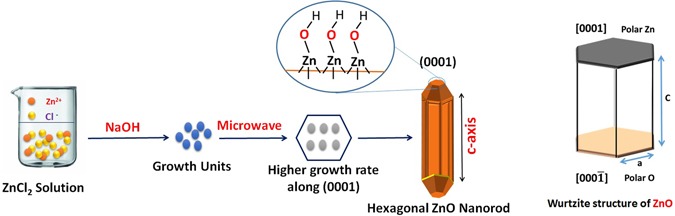
Growth mechanism of ZnO nanorods.

**Figure 5:**
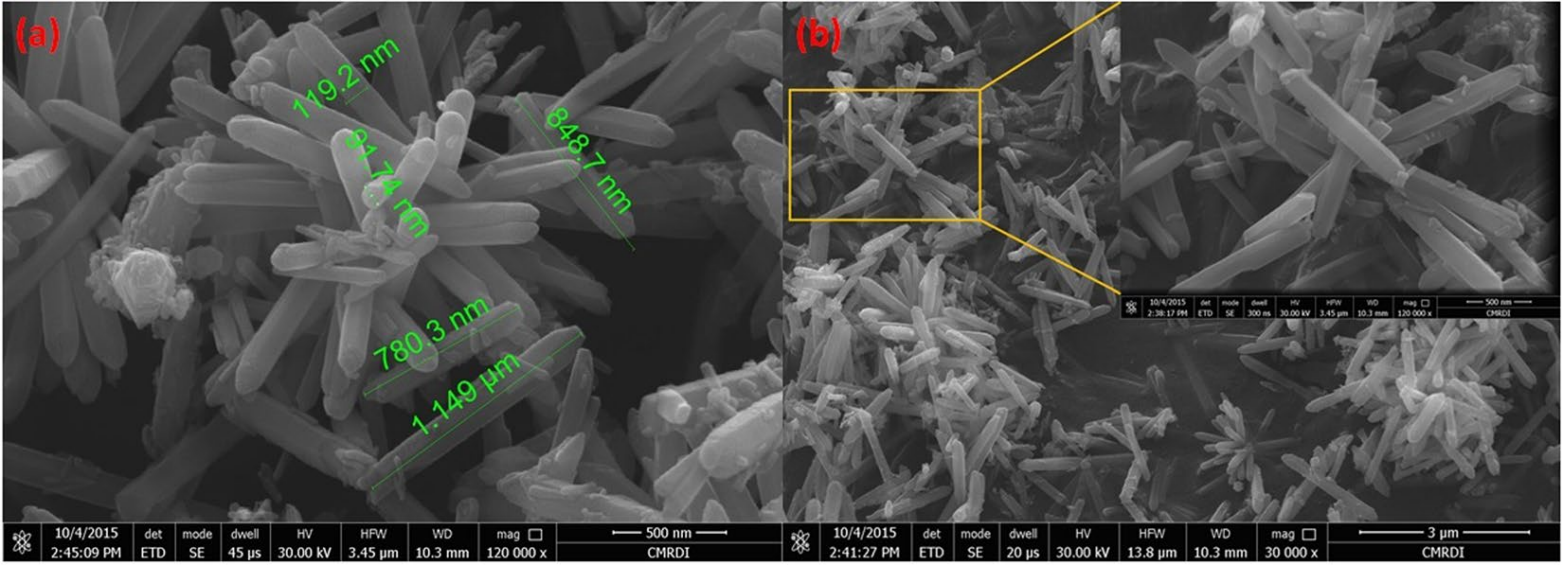
FESEM images of hexagonal ZnO nanorods (**a**,**b**).

**Figure 6:**
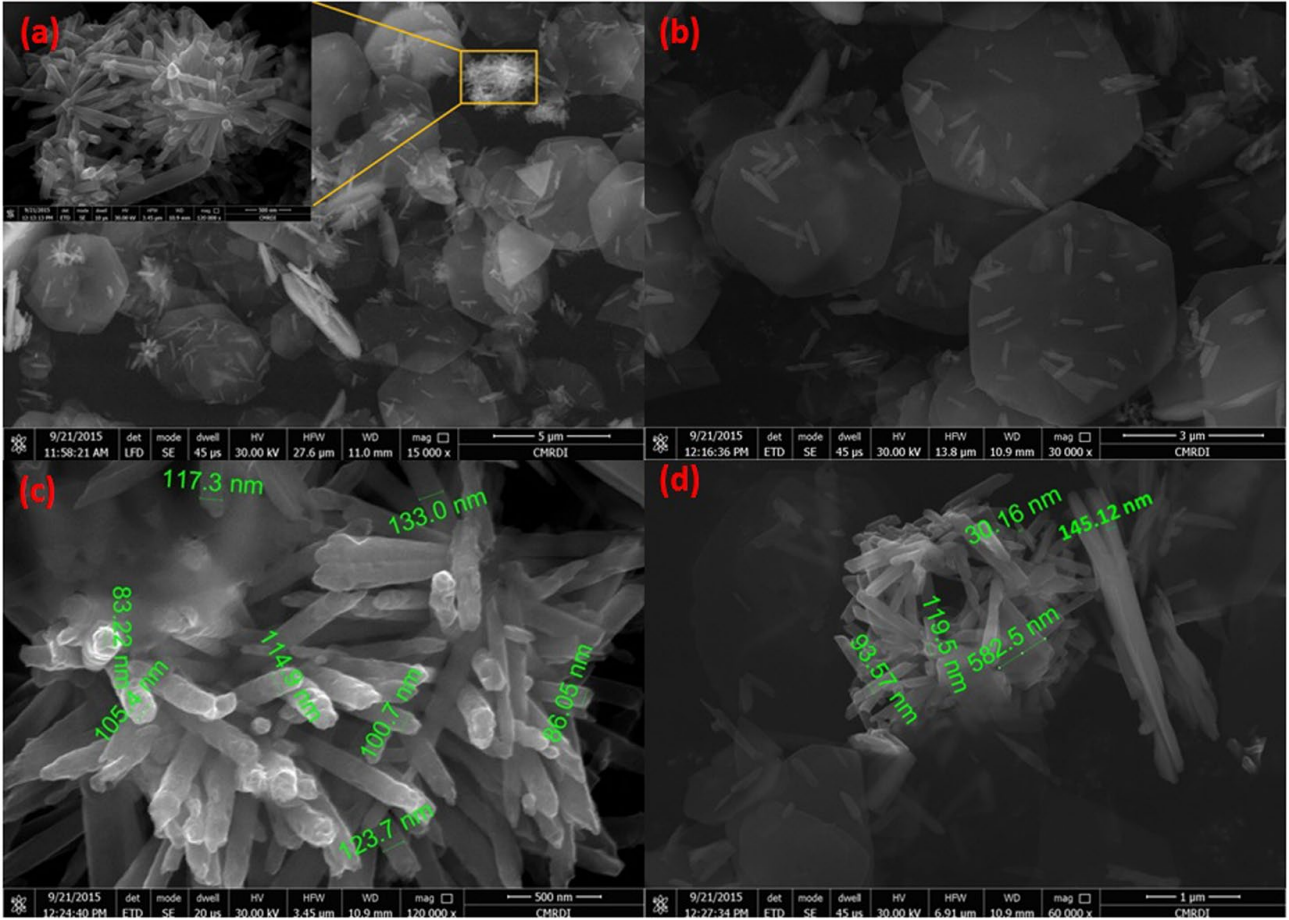
FESEM images of ZnO oxide nanoflower over nanoplatelets (**a**), dispersed ZnO oxide nanorods over nanoplatelets (**b**), collection of ZnO oxide nanorods (**c**), and ZnO oxide nanocage over nanoplatelets (**d**).

Hexagonal zinc oxide (ZnO) nanorods were synthesized by microwave assisted technique. Schematic diagram depicting the growth mechanism of ZnO nanorods is shown in Fig. 4^105^. It is well known that, the preferential growth of the polar crystal of ZnO nanostructure is along c-axis [(0001) direction], in which the tip of the nanorod is terminated by Zn^105^. Nanorod morphology is obtained because the higher growth rate along (0111) than that along (1010) direction^105^. The FESEM observations reveal the morphology transition of ZnO nanorods “pencil-like tip” to the nanoplatelets grafted molybdenum oxides, besides the formation of different ZnO nanostructures by the end of reaction. The reaction involved treatment of ZnO nanorods with phosphomolybdic acid (PMA) under ultra-sonication for several minutes in atmosphere. The starting ZnO nanorods have a diameter ranging from 80 to 150 nm, a length ranging from 500 nm to few micrometers, with an average aspect ratio of about 4–10. Other ZnO nanorods with lower dimensions are also observed during FESEM inspection as shown in Fig. 5. The nanoplatelets have a width up to several micrometers, and thickness nearly about 100–150 nm. Some nanoplatelets with lower dimensions than the predominant ones were also formed, that may have morphological transition from the small ZnO nanorods. Sheets and cracked plates are due to the power of sonication (500 W) as shown in Fig. 6(a). Additionally, ZnO nanorods that formed over the nanoplatelets are either separated or a cluster of agglomerated rods that form flower or cage-like structures. FESEM observation also revealed their dimensions as follow: a diameter in the range of 30–50 nm and length ranging from 500 nm to few micrometers, as shown in Fig. 6. Additional, SEM images represent statistical distribution of the composite components and illustrate their dimensions through the entire sample are in (Supplementary Figure S4). Zeta sizer measurements were used to determine the size distribution (Supplementary Figure S5).

**Figure 7:**
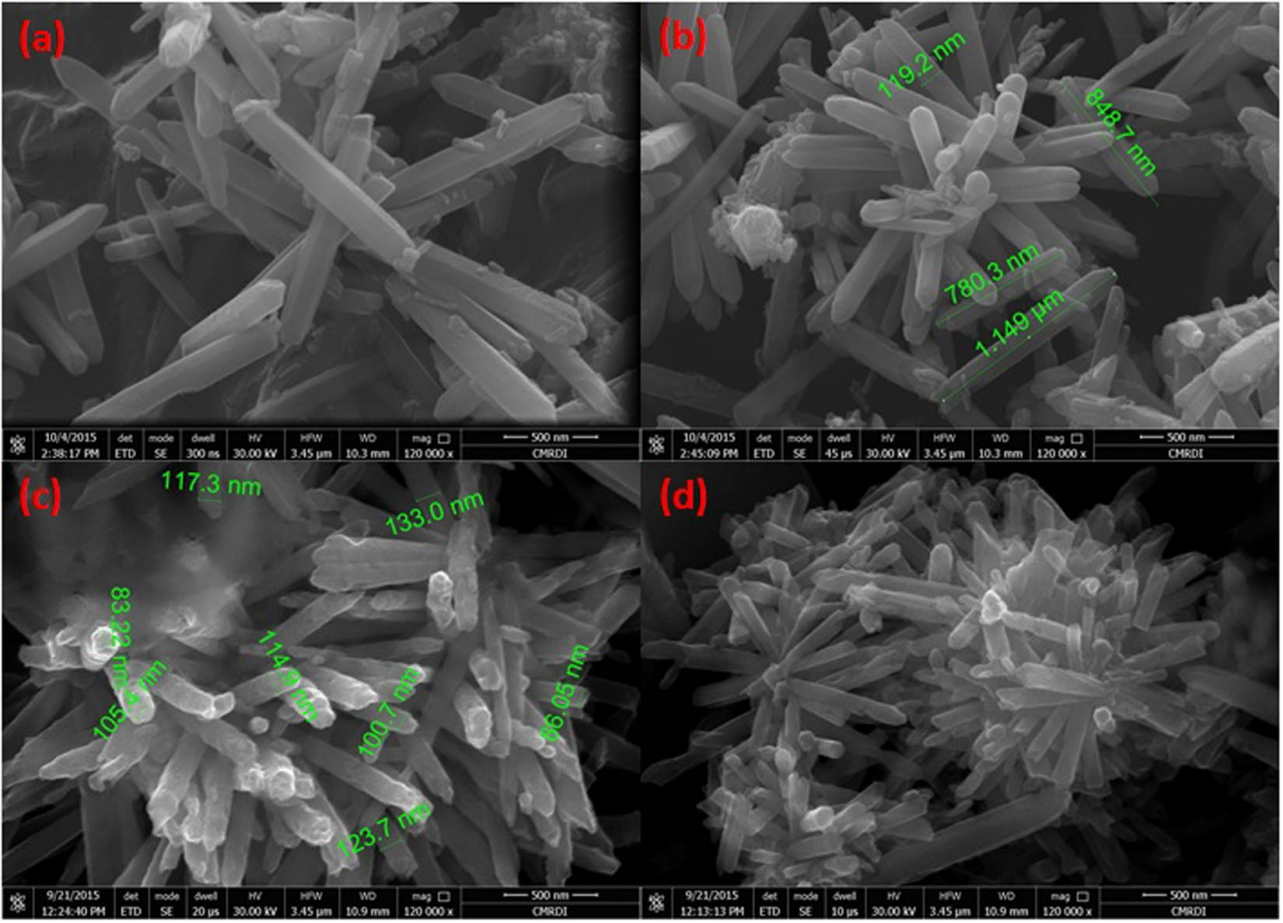
FESEM images of hexagonal ZnO nanorods ‘with pencil like tip’ before reaction (**a**,**b**), and deformed (chemically etched) ZnO nanorods after reaction that has not self-assembled to nanoplatelets (**c**,**d**).

Another observation is the deformation of the outer hexagonal shape of ZnO nanorods that has not self-assembled to nanoplatelets as shown in Fig. 7. This may be attributed to the effect of high acidity of POM solution (chemical etching)^106, 107^ for a short time before being depleted due to consumption of POM anions by catalytic deposition of Mo_8_O_23_-MoO_2_ over ZnO nanoplatelets. Besides, the acidity of POM solution that caused dissolution of outer hexagonal shape, the high temperature (nearly 100 °C) may causes a thermal expansion that will be discussed while explaining the supposed mechanism.

**Figure 8:**
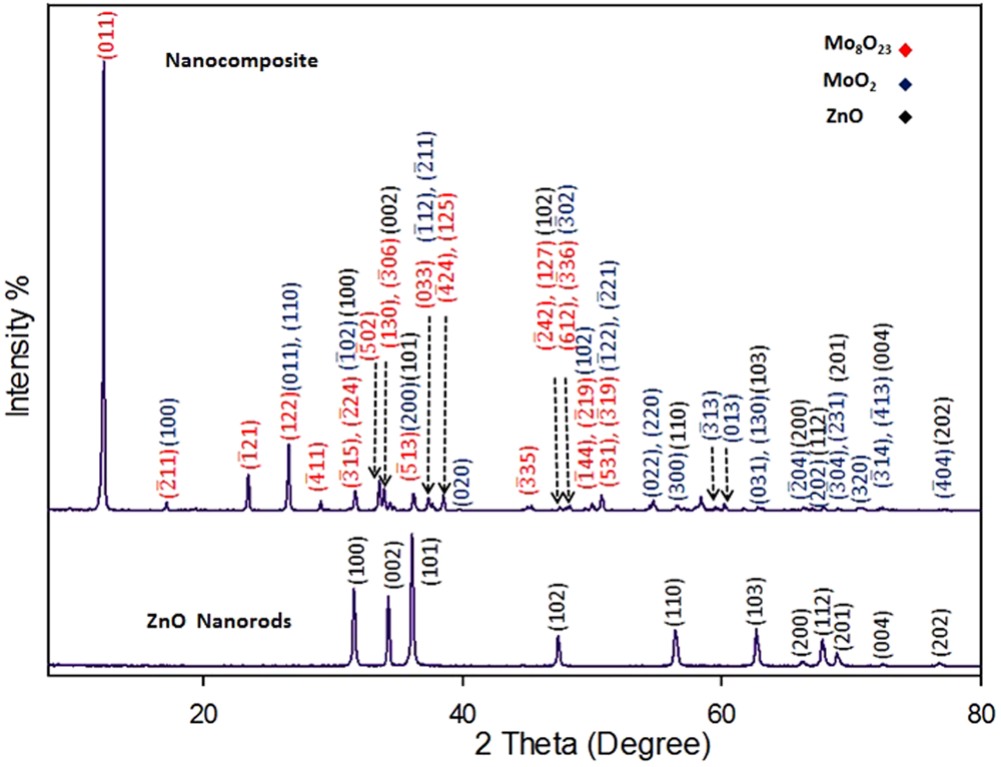
XRD patterns for ZnO nanorods and the nanocomposite (decorated ZnO nanoplatelets grafted Mo_8_O_23_-MoO_2_ mixed oxide).

XRD spectra are recorded for ZnO nanorods and the nanocomposite, which revealed the crystallinity of both materials and the formation of two types of molybdenum oxides. An ICCD card (No: [01-076-0704]) matched well with all peaks of hexagonal ZnO nanorods. Regarding the nanocomposite, all of these ICCD cards (No: [04-005-4566]), (No: [04-055-4547]) and (No: [01-076-0704] which refer to Mo_8_O_23_, MoO_2_ and ZnO, respectively match well with the XRD peaks. The highest peak at 2θ = 12.278° represents the main characteristic peak of the monoclinic Mo_8_O_23_ (011). The other intense peak at 2θ = 26.479° is characteristic for the monoclinic MoO_2_ (011) and (110) orientation. Notice that, the intensity of ZnO characteristic peaks significantly decrease and other identified peaks are detected for all of the composite components as shown in Fig. 8. The remarkable high intensity of (011) Mo_8_O_23_, indicates the predominance of Mo_8_O_23_ over MoO_2_ species.

## Supposed Mechanism

**Figure 9:**
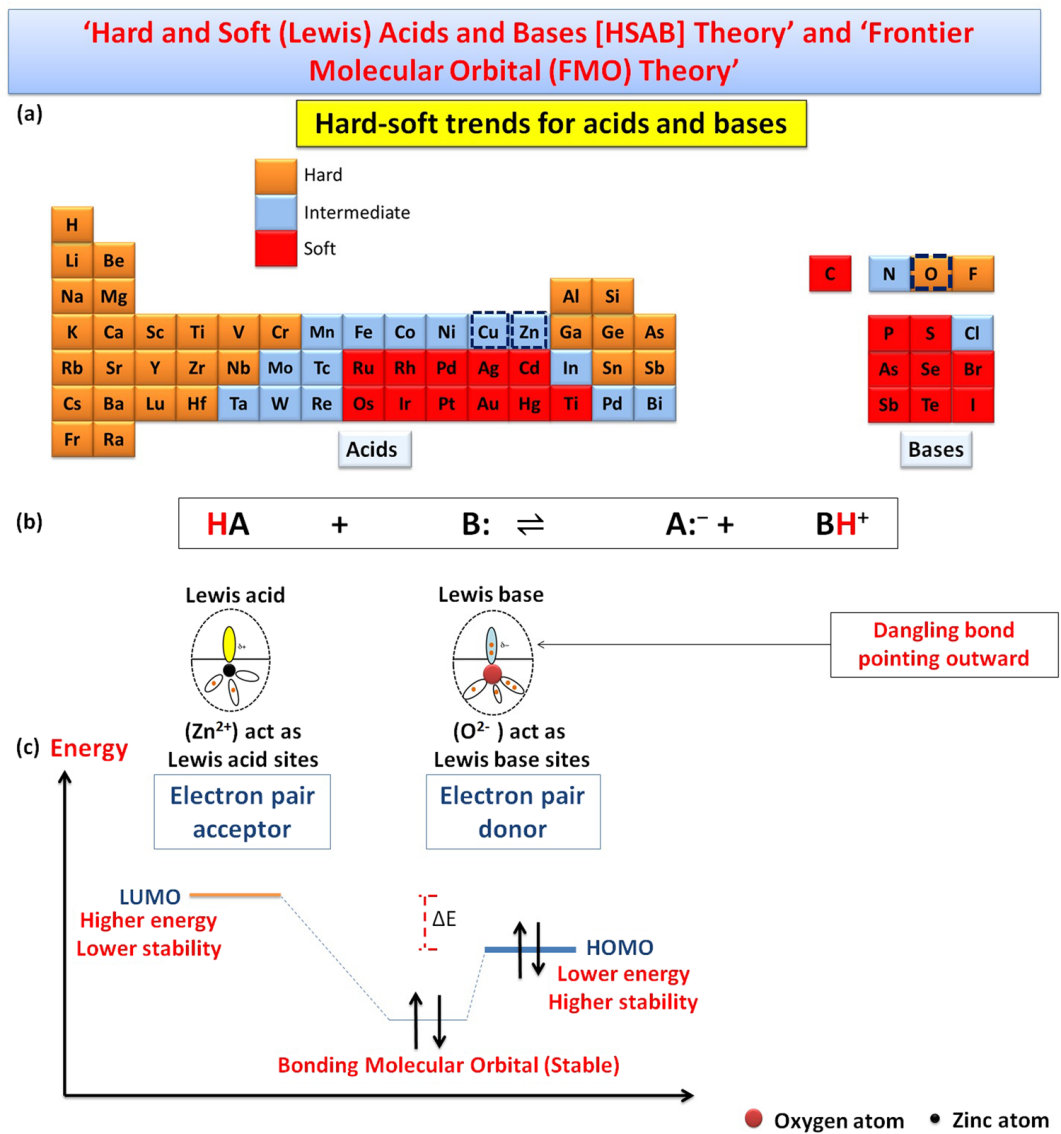
Shows classification of elements as (acids and bases) according to HSAB theory (**a**), behavior of metal cations (LUMO: Zn^2+^) and ligand (HOMO: O^2−^) according to HSAB theory (**b**), and the interaction between LUMO of acid cation and HOMO of base ligand according to FMO theory.

**Figure 10:**
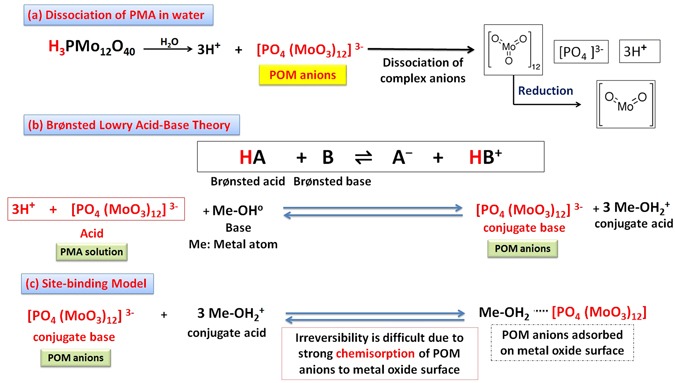
Shows dissociation equation for phosphomolybdic acid (PMA) in water (**a**), the behavior of hydroxyl group on metal oxide surface as Brønsted base site (accept protons) (**b**), and the adsorption of (POM anions) to amphoteric metal oxide surface according to site-binding model (**c**).

**Figure 11:**
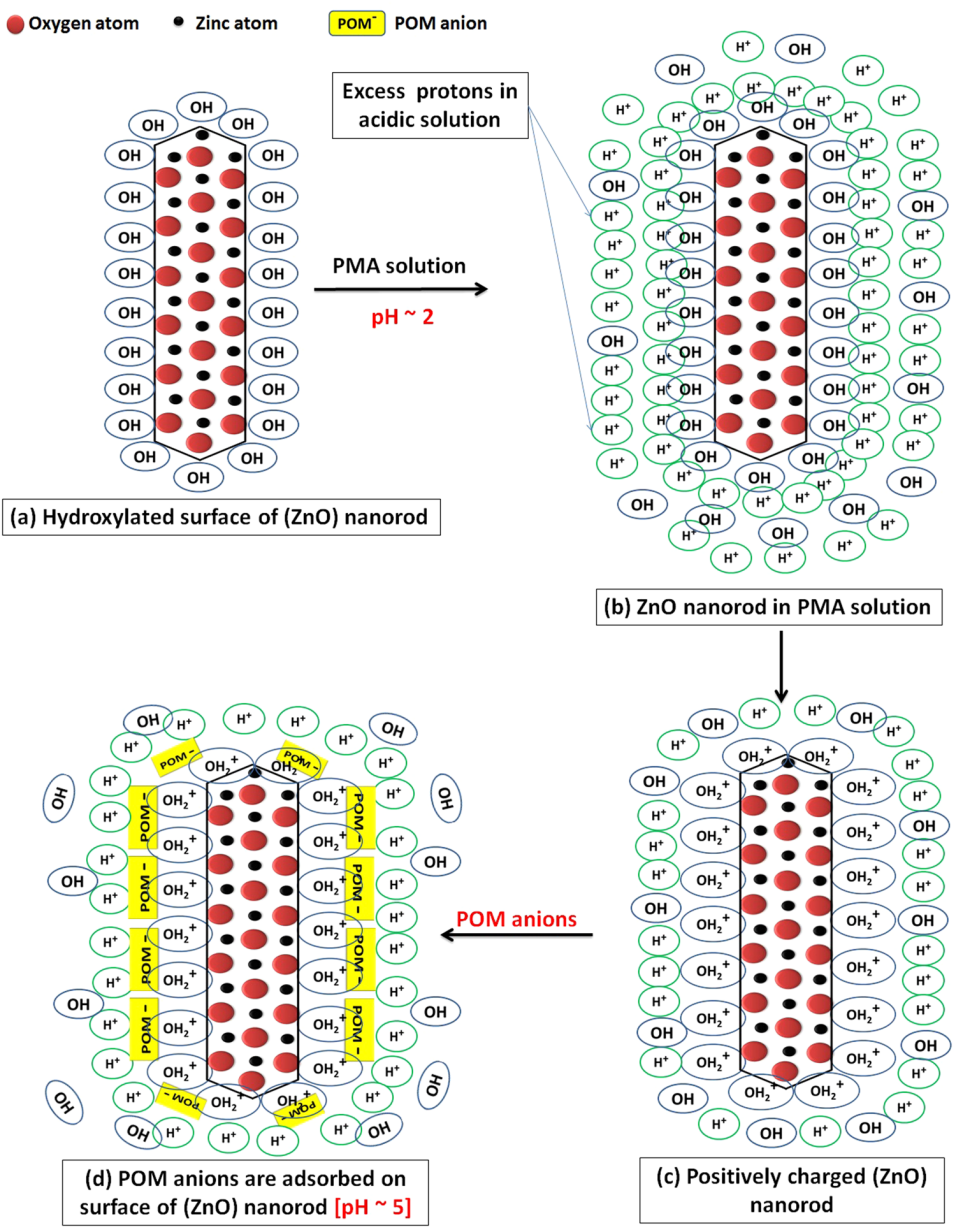
Schematic illustration for the adsorption of protons, then (POM anions) on ZnO surface at early stage of reaction.

We are going to propose a mechanism for the morphology transition of zinc oxide (ZnO) nanorods to nanoplatelets grafted Mo_8_O_23_-MoO_2_ mixed oxide. Through the article, we will postulate a series of selection rules (SRs) which account for the ability of ZnO to experience morphology transition among other binary compounds (metal oxides), and demonstrate theoretical arguments for the ability of other binary compounds to follow the same manner when react with POMs. Regardless of the fact that there is no general theory that serves to determine surface acidity or basicity, acid/base theories account very well for surface chemistry of solids^108^. Here, hard and soft (Lewis) acids and bases (HSAB) theory which is widely used in chemistry for explaining stability of compounds, reaction mechanisms and pathways, can help in accounting for the phenomenon of morphology transition as a long with Brønsted-Lowry acid/base theory, frontier molecular orbital (FMO) theory, and site-binding model^109–112^. The surface of most metal oxides is hydroxylated to some extent under normal conditions when water vapor is present^113^. These surface hydroxyl groups are able to serve as Brønsted acid or base sites as they are able to give up or accept a proton^113^. Since elements in periodic table can be classified as acids and bases according to HSAB theory as shown in Fig. 9(a)^114^, we consider that the cationic metal centers (Zn^2+^) act as Lewis acid sites [LUMO: Lowest Unoccupied Molecular Orbital] while the anionic oxygen (O^2−^) centers act as Lewis bases [HOMO: Highest Occupied Molecular Orbital]^108^ as shown in Fig. 9(b). We refer the fusion process that will be discussed in details to the interaction between (LUMO) of cations with (HOMO) of ligand (empty-filled interaction) which produce a bonding molecular orbital as shown in Fig. 9(c)^115^. It is a fact; the closer the interacting orbitals are in energy the greater the stabilizing energy that the pair of electron that occupies those newly formed molecular orbitals will gain^116^. The HOMO-LUMO interaction (Frontier orbital interaction) results in a large drop in energy; as a consequence the formed molecular orbital will acquire a high stability^115^. Hence, we state the first selection rule (SR_1_) as follow *“The appropriate energy difference between LUMO (acid orbital) and HOMO (base orbital) may account for ability of ZnO to experience morphology transition when react with POMs under specific conditions”*. Acid/base sites are quite important in determining the catalytic activity of metal oxides^108^. When ZnO nanorods is added to PMA solution (pH ~2), the hydroxyl groups adsorbed to ZnO surface act as Brønsted bases in this highly acidic medium and can accepting protons according to site-binding model as illustrated in Figs 10(b) and 11 ^109, 111, 112^.

Amphoteric ZnO surface becomes positively charged by accepting protons, and pH increased remarkably near IEP of ZnO (pH = 5). This ensures the fact that ZnO nanomaterials surface at a certain pH value is positively charged. This pH value is known as the isoelectric point (IEP)^117^. For metal oxides, at pH values above the IEP, the predominate surface species is Me-O^−^, while at pH values below the IEP, Me-OH^2+^ species are predominate^118^. For ZnO the isoelectric point lies within the pH range (8.0–10.0)^119, 120^. Phosphomolybdic acid dissociates in solvent (water) to complex anions [POM anions (Molybdate): [PO_4_ (MoO_3_)_12_]^3-^] and protons as shown in Fig. 10(a). Polyoxomatalate anions (POM anions) are adsorbed strongly to the surface of ZnO nanorods as they exhibit a strong chemisorption activity, which is enhanced by the force of the sonication, together with the expected electrostatic attraction with positively charged ZnO nanorods as illustrated in Fig. 10(b and c).

Adsorption affinity of POM anions towards surface of metal oxides is expected to vary according to the ability of metal oxides to acquire a positive charge when immersed in PMA solution^112^. This depends up on the IEP of metal oxides. Hence, taking in consideration these two facts; firstly, at pH values below the IEP, OH^2+^ species are predominate, and secondly, our reaction is taking place at highly acidic medium (pH~2); *we* state the second selection rule (SR_2_) as follow; *“The suitable isoelectric point (IEP) which locates in basic region (8–10) account for the tendency of ZnO to experience morphology transition when react with POMs, as this guarantees the predominant of OH*^*2+*^ *species which attract POM anions to metal oxide surface”*. We can manipulate the adsorption behavior of metal oxides by varying the acidity of PMA solution which depends on the concentration of PMA. Zeta potential measurements of pure ZnO and the nanocomposite confirmed that ZnO nanorods are surrounded with positively charged groups when it is immersed in a solution that has pH value lower than its IEP. Reduction of the positive charge after complete reaction is due to neutralization by oppositely charged PMA anions as shown in Fig. 10(c) (Supplementary Figure S6).

**Figure 12:**
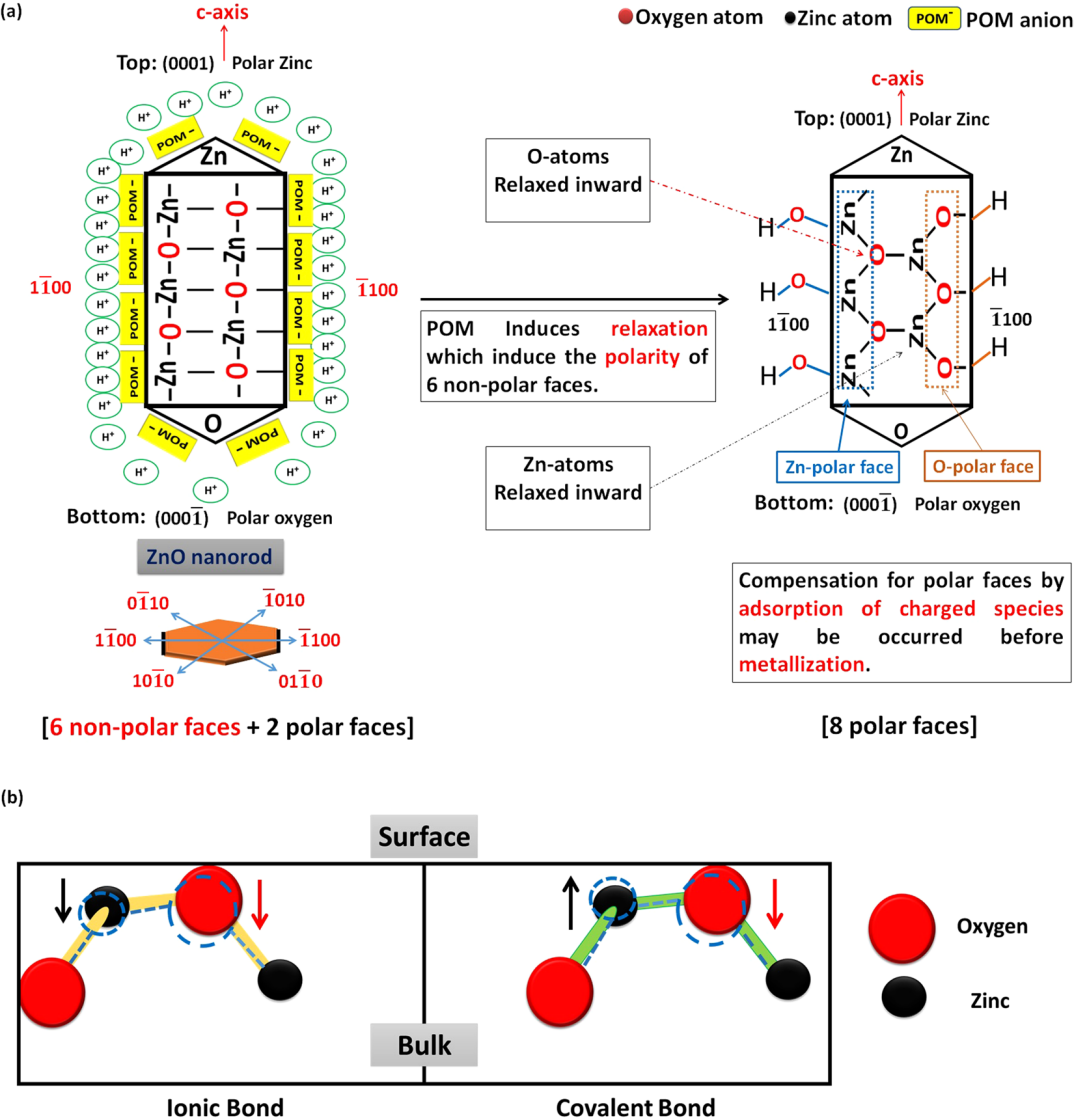
Schematic illustration for the effect of (POM) anions adsorbed on ZnO surface on surface relaxation and polarity (**a**), and the expected relaxation modes [doted lines indicate the position of atoms after relaxation process] (**b**).

Another phenomenon that contributes significantly in the explanation of surface chemistry for transition metal oxides is the surface polarity^121^. This term refer to “the status of the surface plane whether it contains a stoichiometric ratio of cations (Zn^2+^) and anions (O^2−^) or not, which refer to non-polar or polar surface respectively”. Polar surface exhibits a strong dipole and is less stable than non-polar surface, as the presence of dipole moment increase the surface Gibbs energy^122^. On the basis of ‘the perturbation theory of reactivity’ which state that as a pair of reactants approach one another, their orbitals interact and begin to undergo a “perturbation”, we conclude that; the adsorption of acid to an oxide surface perturbs the neighboring acid-base sites. This perturbation inevitably induces the relaxation of the surface^123^ as shown in Fig. 12(a).

Depending on the aforementioned statement, we conclude that the adsorption of POM anions to ZnO surface perturbs the neighboring (Zn^2+^) and (O^2−^) sites. As a consequence, surface relaxation occurs which induce polarity of the six non-polar faces for ZnO. As reported by Tasker *et al*.^124^, polar surfaces are intrinsically unstable, and they can attain stability by various stabilization processes. For instance, if charge is transferred from the O-terminated to the Zn-terminated surface, partially filled bands result^125^, which is known as surface metallization (fusion of nanorods)^126^. Other stabilizing processes are surface reconstruction^127, 128^ or a saturation of the surface with hydroxyl groups via the adsorption of hydrogen or water^125^. This last way may be the case in decorated nanorods that have not experience morphology transition.

The extent and the direction of relaxation depend on the relative contributions of many factors such as ionic size, types of terminated atoms, presence of electron lone pairs on the surface, and the residual influence of covalent bonding^129, 130^. We have a suggestion for the mechanism of surface relaxation induced by POM adsorption. We suppose that, the adsorption of POM anions on ZnO surface has induced the relaxation of O-atoms inward the lattice due to the repulsion between their electric fields created by their electron clouds^131^. Since Zn-cation have a strong polarizing power, the electron cloud of relaxed O-anion is distorted towards Zn-cation, which induce the polarity of bond between oxygen and zinc. Consequently, the covalent character of surface bonds increases (Supplementary – Figure S7).

Due to the covalence character of ZnO ionic bond, Zn-atoms on the surface have two possibilities to acquire surface relaxation. In case of predominance of ionic character, it is possible that both the metal ion and the oxygen ion relax inward, as the restriction on bond directions and lengths are much less severe than in a covalent compound. In case of pure covalent bond, Zn-atoms prefer to relax outwards as shown in Fig. 12(b)^129^. Moreover, the first mode of relaxation was reported as the predominance one for ZnO, which influences the energetic position as well as the dispersion width of the surface dangling bond bands of the occupied O 2p and unoccupied Zn 4 s states^132^. As mentioned before, relaxation of metal oxide surface depends up on many factors such as ionicity which decreases on going to the lower right hand comer of the periodic table. We can predict different mode of relaxation for the surface atoms of different oxides which influence on the ability of surface to acquire polarity, and hence may account for ability of other oxides to experience morphology transition. To sum up, both relaxation modes are expected to occur and the polarity may be induced more by chemical etching of surface atoms by PMA.

On the basis of the aforementioned observations and facts, we propose a mechanism for the phenomenon of morphology transition which involves self-assembly of ZnO nanorods to nanoplatelets simultaneously with deposition of Mo_8_O_23_-MoO_2_ over zinc oxide, as schematically shown in Fig. 12(a). *First step*, involves the chemisorption of POM anions on the surface of zinc oxide (ZnO) nanorods^44^. The adsorption of these anions constitutes the driving force for surface relaxation by disrupting the neighboring atoms, which induce the polarity of the non-polar faces^123^. Consequently, Zn-polar and O-polar faces are formed^121^ which may be equivalent for each single nanorod (i.e, 3 Zn-polar and 3 O-polar faces). In other words, according to ZnO Pourbaix diagram, the pH of POM solution (~2) allows the dissolution/chemical etching of zinc oxide^106, 107, 133^. The observed immediate increasing in pH (reach ~5 within 2 minutes) prevents further etching or complete dissolution of ZnO. This chemical etching causes surface relaxation and induces polarity by removing zinc cations or oxygen anions from the outer surfaces. These etched species are taking part in self-assembly process^132^.

**Figure 13:**
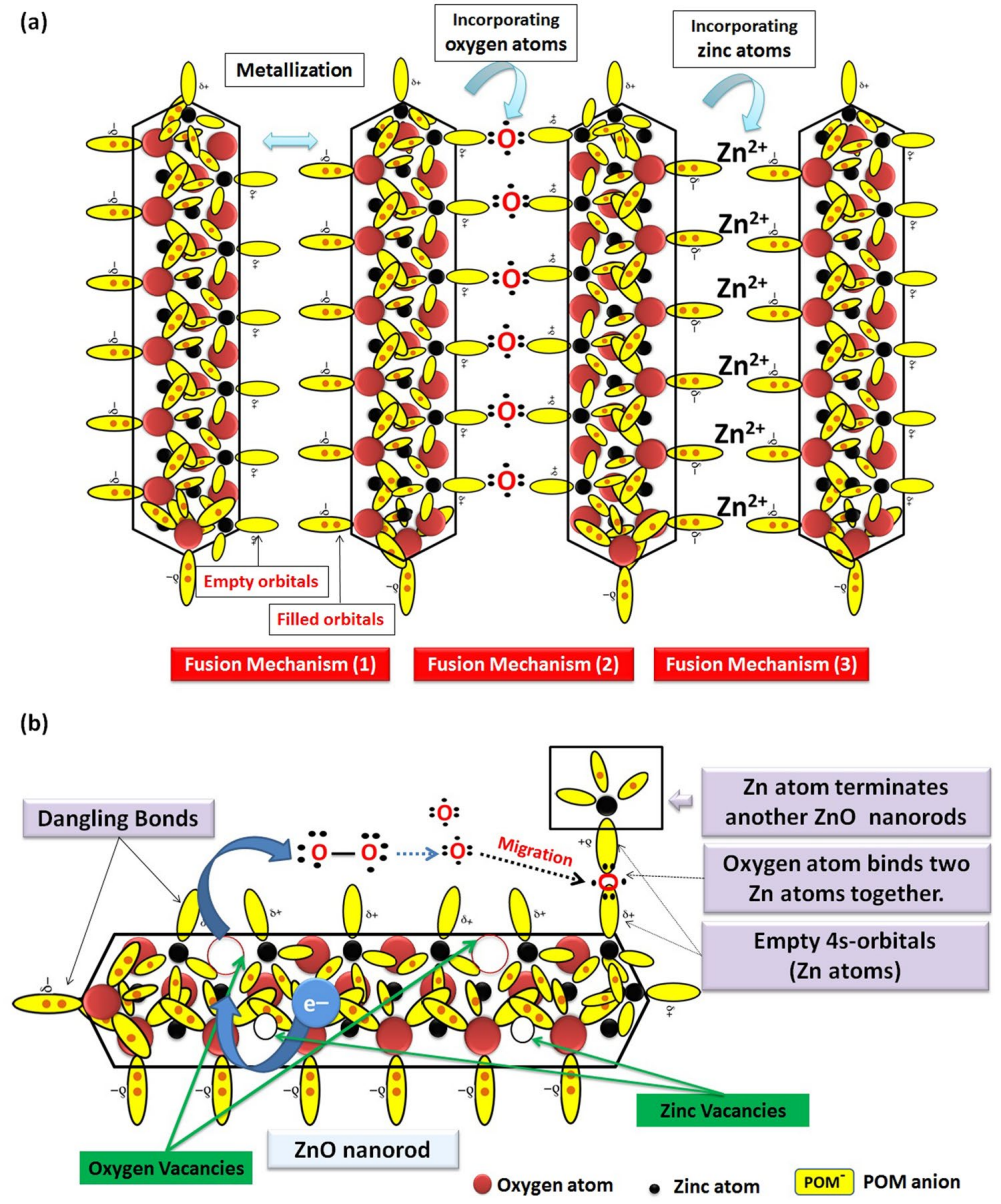
Schematic illustration for the three possible fusion mechanisms of ZnO nanorods in PMA solution (**a**), and schematic illustration for catalyzing the O-O bond cleavage of O_2_ molecules by electron transferred from lattice oxygen atoms to vacancies to the O_2_ molecule (**b**).

Since, metastable surface of ZnO nanorods was brought to un-stable state by surface relaxation induced by POM anions^123^, these nanorods tend to be stable by fusing and forming nanoplatelets^134^. For this *second step*, we assume *three main mechanisms*; the first one, involves fusing of two faces with opposite polarity by bonding Zn-atom to O-atom through their dangling bonds [empty Zn 4s-orbitals and filled O 2p-orbitals] to form bonding molecular orbital with lower energy and high stability. This process is known as metallization^126^ [Fusion Mechanism (1)]. Since, zinc oxide is amphoteric and dissolves easily in acids^135^, which accounts for the chemical etching of ZnO nanorods that produces aqueous Zn^2+^ and O^2−^ at early stage of reaction^106, 107^. From this point of view, we suppose the second and third mechanism which involves fusing of two faces with similar polarity accompanied with incorporating of external atoms into the lattice^130^. On one hand, oxygen anions migrate through surface and incorporate to the lattice by binding to two empty Zn 4s-orbitals (dandling bonds) of two Zn-terminated surfaces resulting in fusion of nanorods [Fusion Mechanism (2)]. On the other hand, zinc cations migrate through surface and incorporate to the lattice by binding to two filled O 2p-orbitals of two O-terminated surfaces resulting in fusion of nanorods [Fusion Mechanism (3)]. Schematic illustration for the three possible fusion mechanisms is illustrated in Fig. 13(a).

This metallization mechanism may be the prevailing one at early stage of reaction, as the concentration of zinc cations and oxygen anions species at this stage is not so high to fuse nanorods together. Moreover, fusion by incorporating oxygen anion is more predominant than that by incorporating zinc cations as the etching rate of oxygen anions is more than that of zinc cations^107^. Long chain-fusion which may produce platelets with extra width was inhibited by reconstruction to triangular-shaped tips^127^ or catalytic deposition of molybdenum oxides over fused ZnO nanorods which hinder the active sites responsible for fusion. After fusion process, the sample weight is duplicated due to catalytic deposition of molybdenum oxides, which was indicated visually by sudden solidification and volume increase (pH: 6.7–7). The reaction continues by further sonication (Supplementary Figure S8).

We expect the formation of defects such as oxygen vacancies (V_O_) due to chemical etching of ZnO nanorods. These oxygen vacancies (V_O_) on the surface of polar ZnO are expected to play a vital role in O-O bond cleavage of atmospheric O_2_ molecules as schematically illustrated in Fig. 13(b)^136–138^. This catalytic process involves electron transfer from lattice oxygen atoms to vacancies to the O_2_ molecule, mediated by the subsurface transition metal cations. The resulting oxygen anions may take part in binding two empty Zn 4s-orbitals (dangling bonds) of two Zn-terminated surfaces resulting in fusion of nanorods [Fusion Mechanism (2)]. This electron transfer through the lattice to the O-vacancies is due to the accumulation of electric charge (Piezoelectricity) in W-ZnO^139^, which induced by mechanical stress resulted from relaxation and expansion of lattice. We still need to do precise experiments at inert atmosphere to study the contribution of atmospheric oxygen to fusion process.

## Catalytic Deposition Mechanism

Depending on the thermodynamic properties of the gas phase we suppose either the formation of OH groups or the formation of oxygen vacancies would be the energetically favorable process for stabilization of top and down surface of ZnO nanoplatelets^125^. Moreover, polar surfaces acquire a certain amount of energy (surface energy), due to the unsatisfied bonds of the atoms at the surface, which known as “dangling bonds”^107, 132, 136, 140–142^. The oxygen atoms at the oxygen-polar surface of ZnO have two electrons in the lone-pair orbital pointing outward from the surface, while the Zn atoms in the (0001) Zn polar surface do not have such filled orbitals^141^. These orbitals are representing the dangling bonds. Moreover, Zn-terminated polar surfaces have high surface energy which explains the experimentally observed high activity of these surfaces than O-terminated polar surfaces towards gas adsorption and surface reactions. The stability of the latter is due to the fact that oxygen is more polarizable than metal ions which lower its surface energy. Anyway, both of them are expected to catalyze the deposition of Mo_8_O_23_-MoO_2_ mixed oxide.

**Figure 14:**
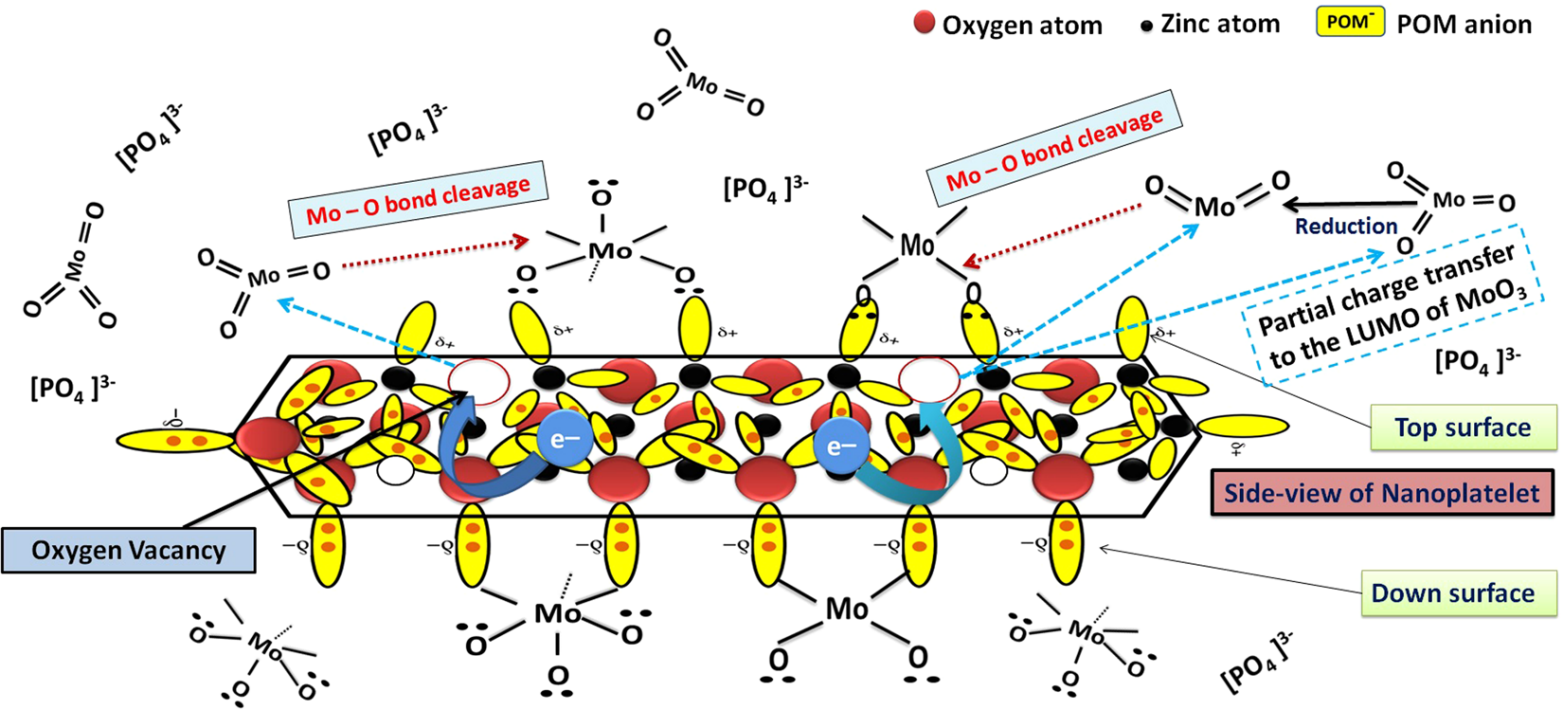
Schematic illustration for the catalytic deposition of Mo_8_O_23_-MoO_2_ mixed oxide over ZnO nanoplatelets.

Figure 14 illustrates schematically a simple mechanism for the catalytic deposition of molybdenum oxides over active ZnO polar surface that has experienced relaxation^143–146^. Regardless of the fact that, Zn-polar surfaces are more active then O-polar surfaces; both of them are expected to take part in this process^136^. After few minutes from starting of reaction, the temperature increases due to sonication power (500 W) and the energy released dissociate the complex anions (POM anions) into (MoO_3_)_12_ and (PO_4_)^3−^. Phosphate anion dissolved easily in hot water. Reduction of a certain amount of (Mo^VI^) to (Mo^IV^) may be attributed to the traces of anions in phosphomolybdic acid used in synthesis (e.g., (Cl^−^: ≤50 mg/kg and SO_4_^2−^: ≤100 mg/kg). A second argument for the mixed valence oxides is the partial charge transfer from the metal oxide surface to the LUMO of the clusters^44^. A third argument is the fact; molybdenum trioxide is dissolved in alkali solution and the simple molybdate anions [MoO_2_^−4^ (Mo^VI^)] anion is produced. Under the influence of pH, other species are formed with lower oxidation state like [Mo_7_O_6_^−24^ (Mo^V^)]^147^. We thought that, all of the aforementioned reasons have worked synergistically, to reduce a portion of (Mo^VI^) to (Mo^IV^). Both of filled and empty dangling bonds on the surface of oxygen and zinc polar faces, start to bind with molybdenum and oxygen forming layer of Mo_8_O_23_-MoO_2_ mixed oxide. Filled d-orbitals pointed outward the surface are expected to take part in this process by trapping MoO_x_ molecules and form bonds with them. Mo_8_O_23_-MoO_2_ deposition shield ZnO from PMA solution and hence, ZnO Pourbaix diagram cannot be considered for the hybrid ZnO nanoplatelets grafted Mo_8_O_23_-MoO_2_ mixed oxide.

**Figure 15:**
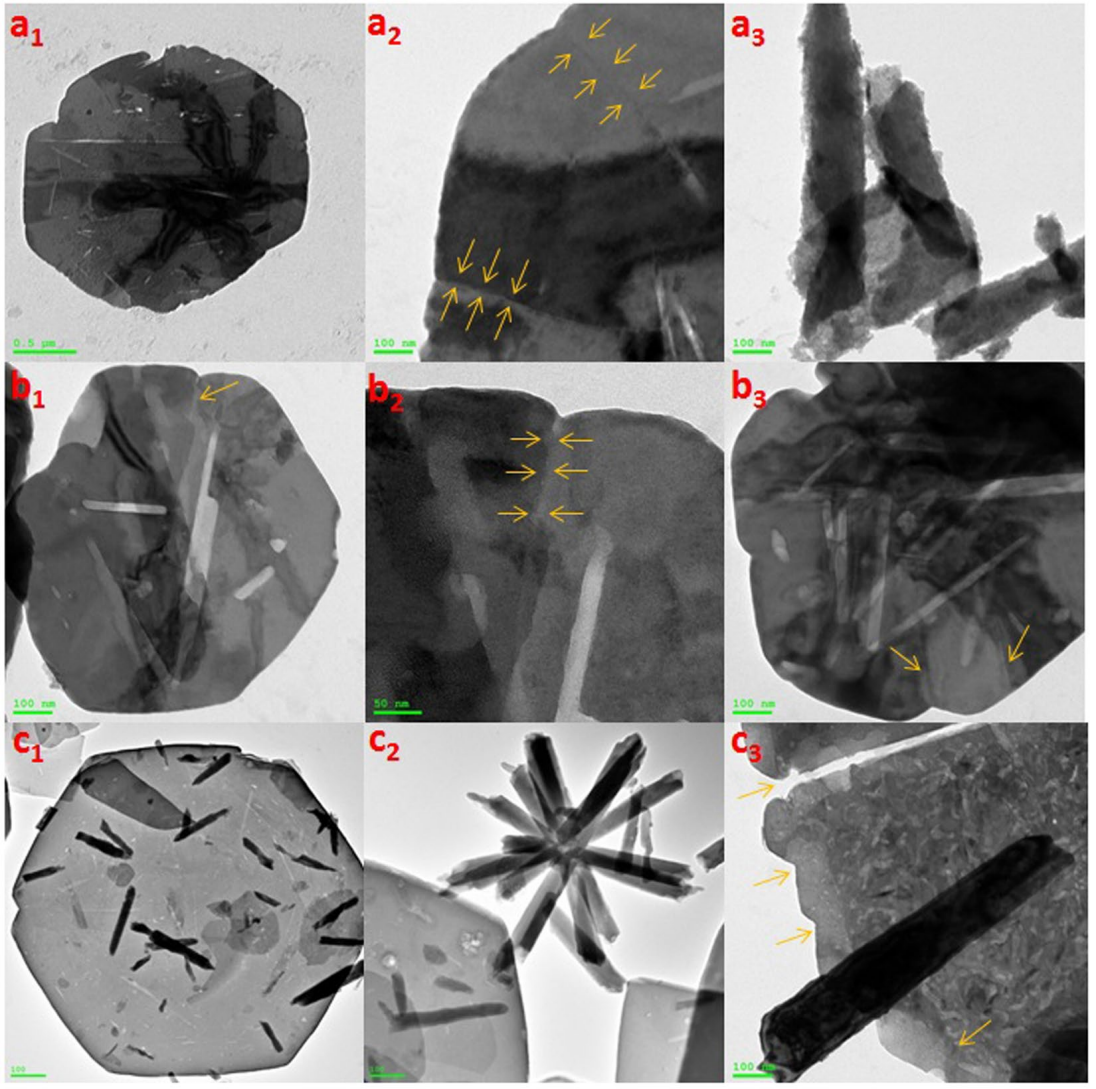
HRTEM of nanoplatelets (**a**_**1**_, **a**_**2**_) with grain boundaries (yellow arrows) and ZnO nanorods are agglomerating to design the decorating ZnO nanostructures (**a**_**3**_), nanoplatelets with grain boundaries decorated ZnO nanorods (**b**_**1**_, **b**_**2**_, and **b**_**3**_), well-shaped nanoplatelets decorated ZnO nanorods and nanoflowers (**c**_**1**_ and **c**_**2**_) and disconnected nanoplatelets (**c**_**3**_) [Samples a, b and c were picked up at 3.5, 8.5, and 15 minutes, respectively].

**Figure 16:**
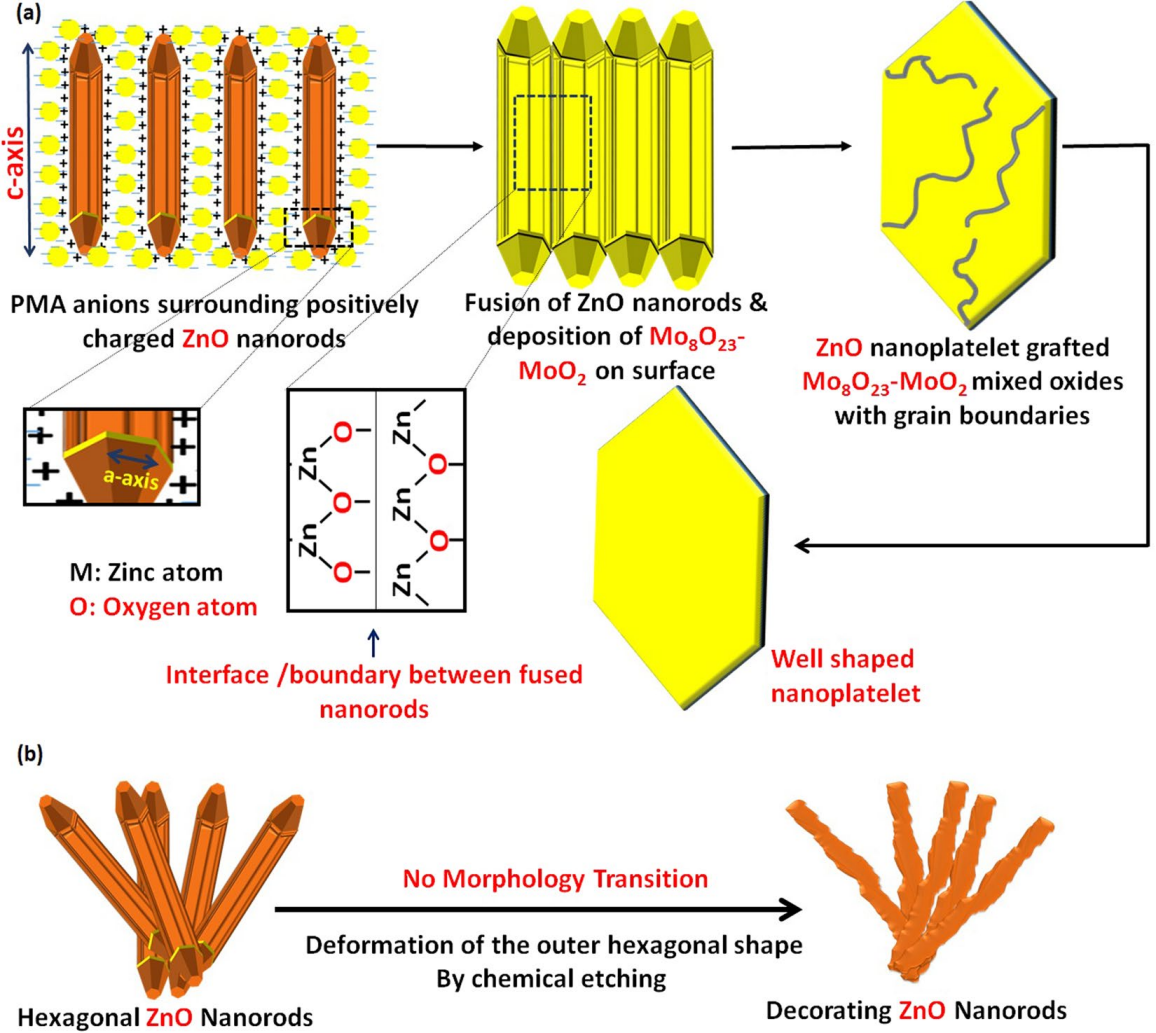
The proposed mechanism for the morphology transition (self-assembly) of hexagonal ZnO nanorods to ZnO nanoplatelets grafted Mo_8_O_23_-MoO_2_ mixed oxide.

We followed up the mechanism of the reaction by HRTEM for three samples of the nanocomposite that were picked up at different intervals (3.5, 8.5, and 15 minutes) from the beginning of sonication, as shown in Fig. 15. HRTEM investigation reveals that hexagonal ZnO nanorods ‘with pencil like tip’ are crosslinked together by PMA anions^148^ and self-assembled to nanoplatelets with grain boundaries as shown in Fig. 15(a_1,2_ and b_1,2,3_). From one perspective, as the temperature increases, crosslinking ZnO nanorods experienced a thermal expansion parallel (${\alpha }_{\parallel }c$) and perpendicular (${\alpha }_{\perp }c$) to c-axis with a difference in factor nearly equal to 1.6, as the thermal expansion coefficients (α) are strongly direction-dependent^149^. Under this condition, the thermal energy that was produced by sonication was not enough for complete fusion and some nanorods can be disconnected as shown in Fig. 15(c_3_). Deposition of Mo_8_O_23_-MoO_2_ mixed oxide over fused-thermally expanded ZnO nanorods guarantees the adhesion of its hexagonal structure and hinders the grain boundaries as shown in Fig. 15(c_1,2_)^150, 151^. More detailed HRTEM images are in (Supplementary Figure S9). A brief schematic illustration for proposed morphology transition mechanism is shown in Fig. 16.

**Figure 17:**
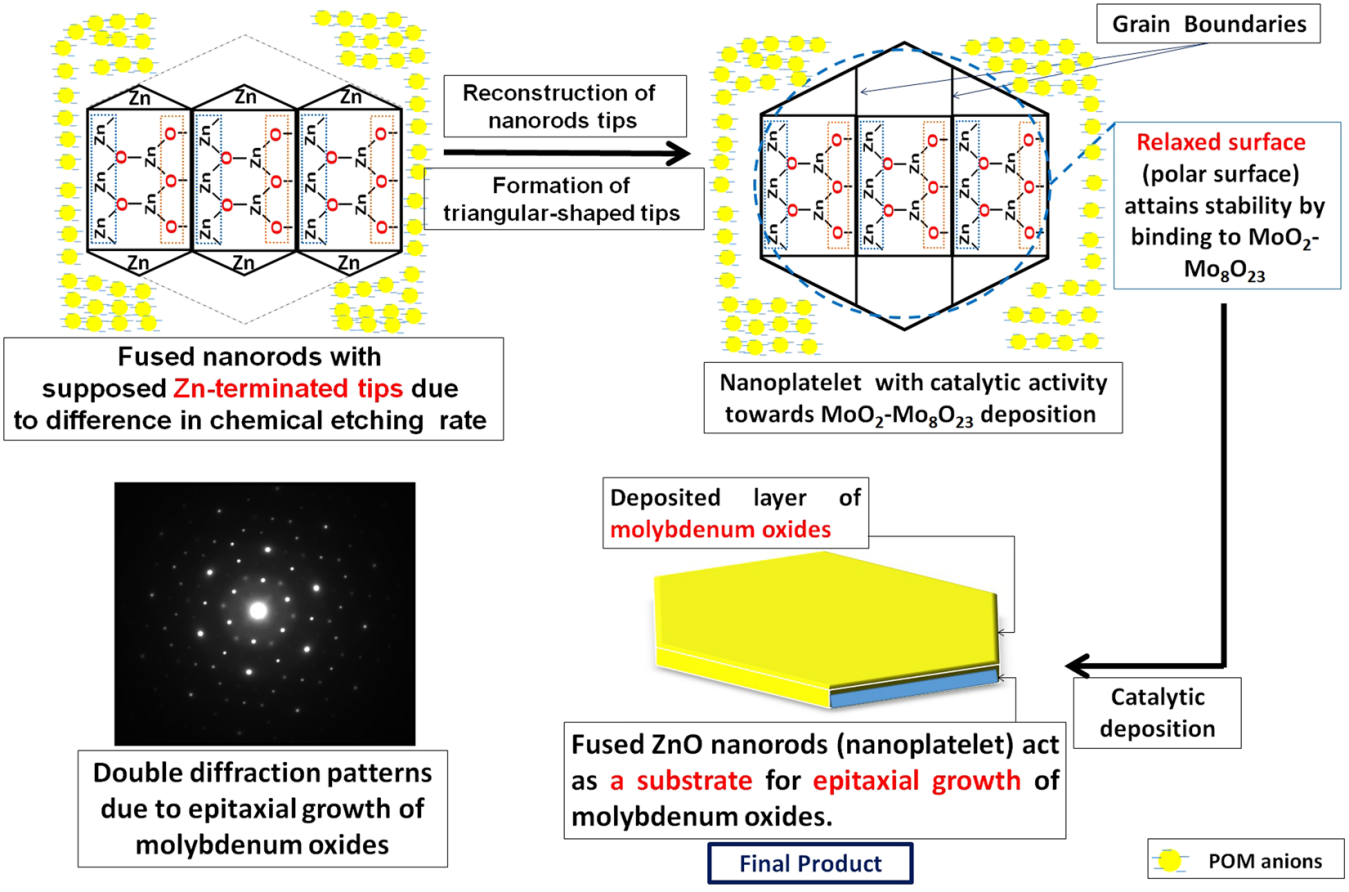
Schematic illustration for reconstruction of nanorods tips to triangular structure and the epitaxial growth of molybdenum oxides over ZnO nanoplatelets.

Mo_8_O_23_-MoO_2_ mixed oxide is supposed to deposit from PMA solution on the surface of fused ZnO nanorods (nanoplatelets). Hence, PMA is supposed to act as an adhesive agent (catalyst) for merging and self-assembling ZnO nanorods to nanoplatelets without diffusion of molybdenum oxides through ZnO crystals. The reduced form (Mo^IV^) along with the (Mo^VI^) species is oxidized in the air to Mo_8_O_23_-MoO_2_ mixed oxide. Furthermore, we suppose the epitaxial growth^152^ of Mo_8_O_23_-MoO_2_ mixed oxide over fused ZnO nanorods. The electron diffraction inspection for ZnO grafted Mo_8_O_23_-MoO_2_ nanoplatelets reveal double diffraction patterns that indicate either topotaxial or epitaxial growth. The latter growth mechanism is more recommended as it involves the deposition of phase A (Mo_8_O_23_-MoO_2_ mixed oxide) on the surface of a crystal of phase B (ZnO) from solution, without diffusion through or reaction with fused ZnO nanorods. In our case, we suppose that fused ZnO nanorods (nanoplatelets) acts as a substrate over which a thin layer of molybdenum oxides is deposited from solution. This supposed mechanism is similar to that of the aqueous chemical solution deposition (CSD) process for epitaxial growth of complex oxides. The similarity is represented in exposing the precursor to excess amount of water, and using of water soluble precursors^153^. As usual in epitaxial growth, the crystals of deposited layer (Mo_8_O_23_-MoO_2_) and substrate (ZnO) have a common crystallographic plane and there is also azimuthal orientation in this plane so that the structures are oriented with respect to each other in three dimensions, resulting in double diffraction patterns^152^ as shown in Fig. 17. This mechanism still needs more evidences.

We expect high etching rate on O- surface because of the dangling electrons on this surface and their tendency to react with electron-seeking agents in the etchant. In contrast, a slow etching rate of the Zn- surface is expected due to the absence of any dangling electrons on this surface^107^. This expected difference in etching rate may lead to fused nanorods with similar polar tips (Zn-polar surfaces). Since, Zn-terminated ZnO (0001) surface acquire a reconstruction stabilization mechanism, result in formation of triangular-shaped tips^127^ as shown in Fig. 17. Hence, we attribute the hexagonal shape of nanoplatelets to this reason. The compensation of polar faces by adsorption of charged species may be occurred before metallization.

**Figure 18:**
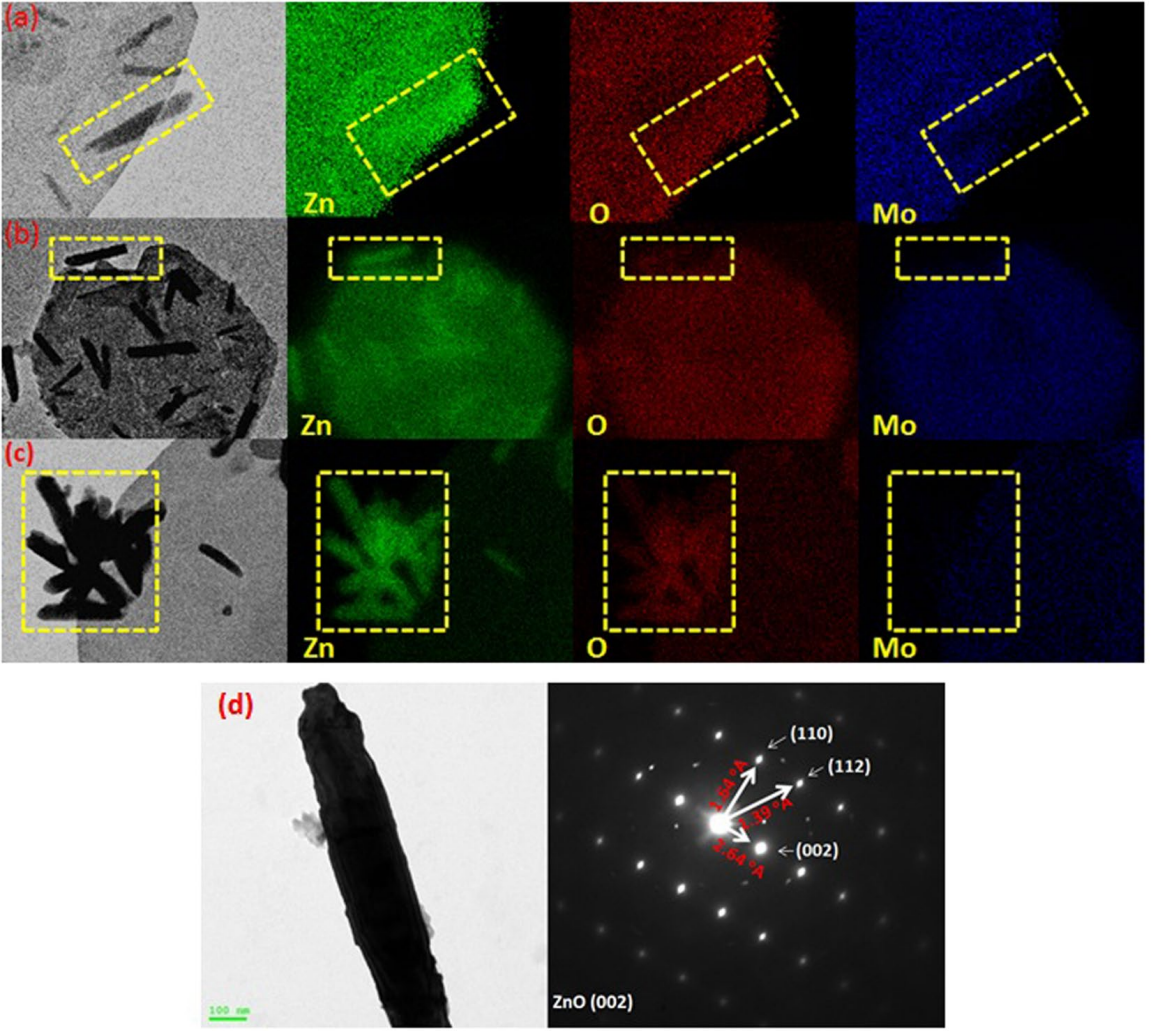
EDX mapping of sample picked up at (**a**) 3.5 minutes, (**b**) and (**c**) 15 minutes after starting of the reaction, and (**d**) HRTEM image of selected ZnO nanorod (left) & its diffraction pattern (right).

**Figure 19:**
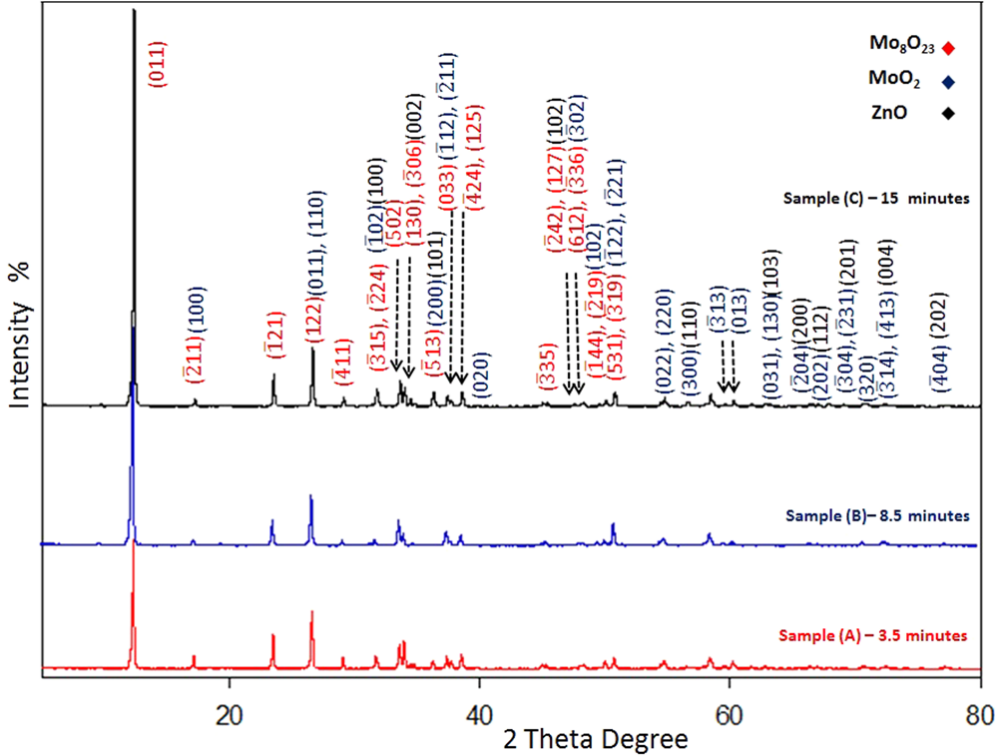
XRD Patterns for three samples of the nanocomposite that were picked up at different intervals (3.5, 8.5, and 15 minutes) after starting of reaction.

EDX mapping supported the growth mechanism by revealing the well distribution of molybdenum through the whole surface along with other elements (O and Zn) as shown in Fig. 18(a–c). XRD spectra were recorded for the three samples of the nanocomposite that were picked up at three different intervals [3.5, 8.5, and 15 minutes] during the reaction. As shown in Fig. 19, the intensity of the peaks increased when we approach the end of reaction. The remarkable intensity of the characteristic peak (011) of Mo_8_O_23_, indicate the predominance of Mo_8_O_23_ over MoO_2_ species during all stage of reaction. Figure 18(d) shows the selected area electron diffraction (SAED) patterns of a selected decorating ZnO nanorod. The results show a good crystalline quality of the obtained material, which is consistent with the XRD result and indicates that the nanorods have a single crystal hexagonal structure. There is only small deviation of diffraction patterns from c-axis, which may be due to the effect of acidity and temperature on the lattice as this is the diffraction patterns for ZnO nanorod that has not self-assembled to nanoplatelet.

Further characterizations to ensure the chemical composition of the composite were carried out using energy-dispersive X-ray spectroscopy (EDX) and EDX mapping which reveal that all decorated nanostructures are either pure ZnO nanorods or ZnO nanorods that were collected closely by few amount of molybdenum oxides to form flower and cage like structures as shown in Fig. 7(a–c). The ratio of Zn to Mo of a selected nanorod area is (99.6:0.4); while the ratio of Zn to Mo in selected nanoplatelets area is nearly (77:23) (Supplementary Figure S10).

**Figure 20:**
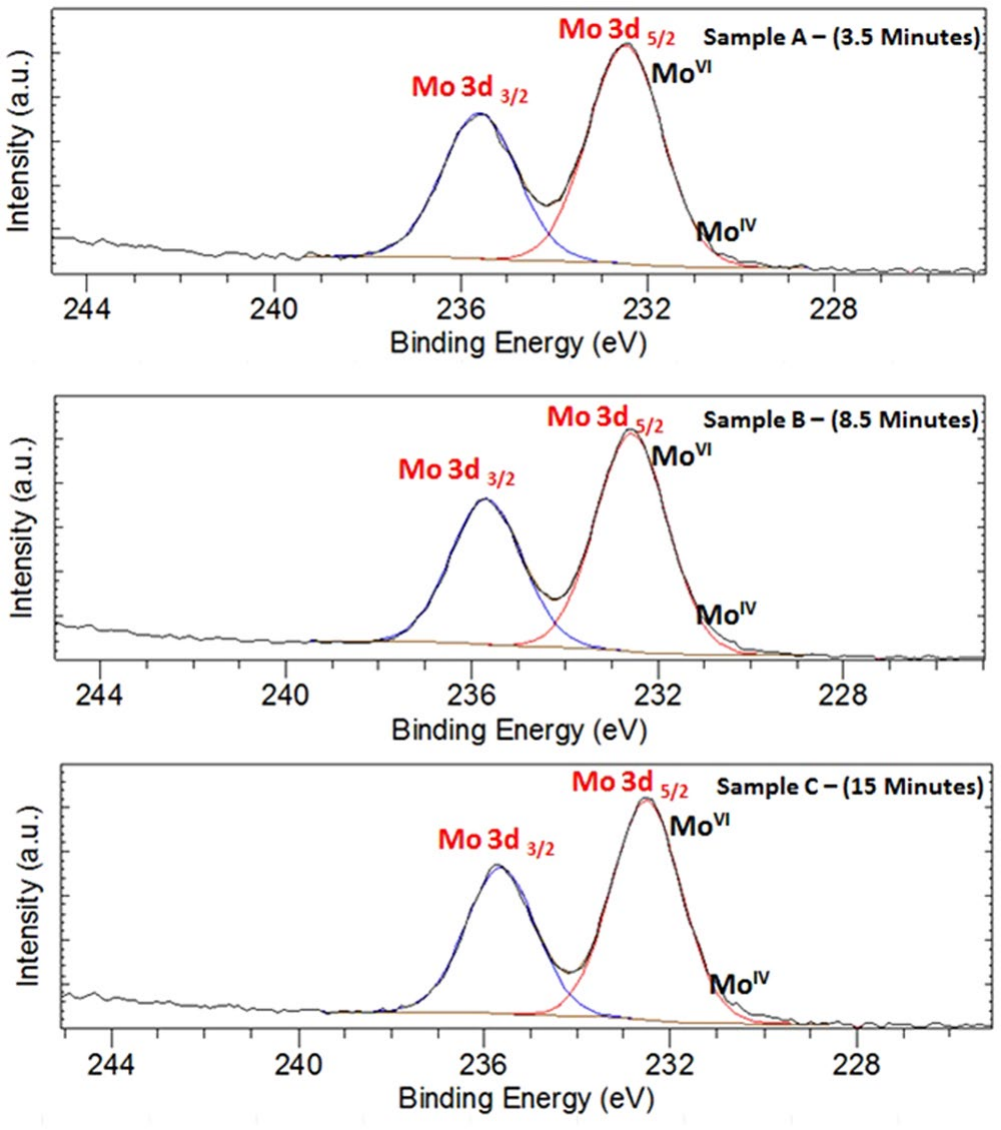
Mo 3d XPS spectra for samples picked up at (**a**) 3.5 minutes, (**b**) 8.5 minutes, and (**c**) 15 minutes after starting of the reaction.

**Figure 21:**
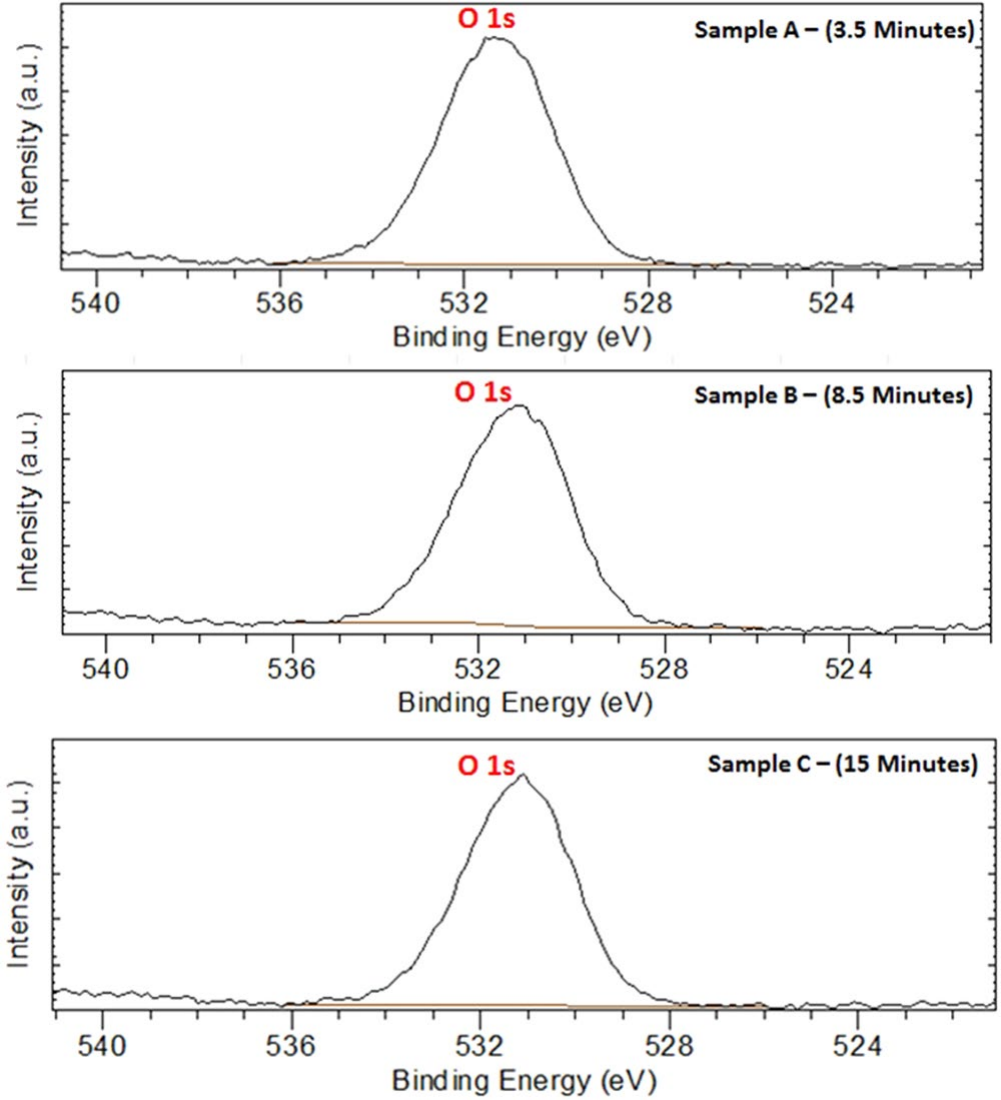
O 1 s XPS spectra for samples picked up at (**a**) 3.5 minutes, (**b**) 8.5 minutes, and (**c**) 15 minutes after starting of the reaction.

**Figure 22:**
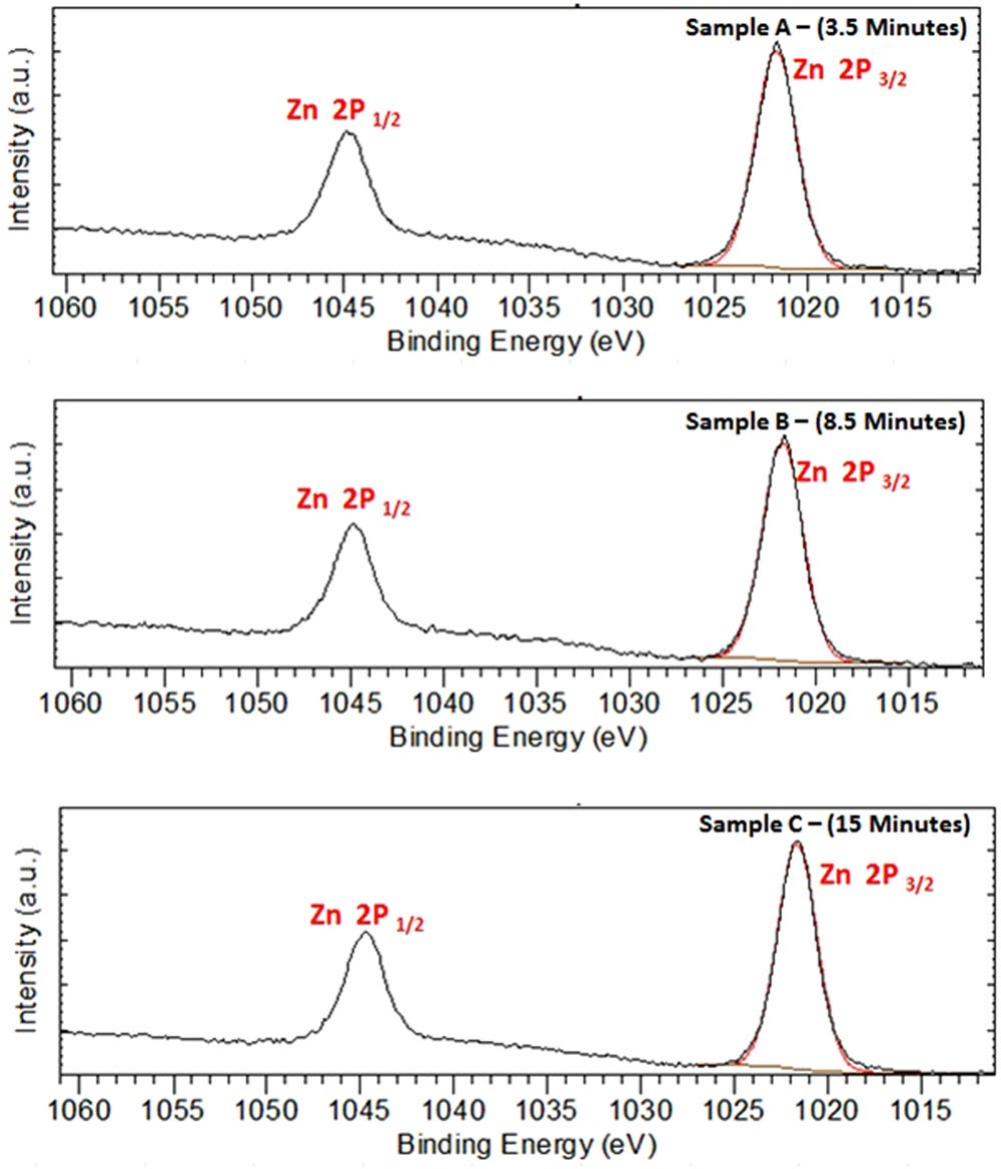
Zn 2p XPS double spectra of ZnO for samples picked up at (**a**) 3.5 minutes, (**b**) 8.5 minutes, and (**c**) 15 minutes after starting of the reaction.

Figure 20 displays the Mo 3d XPS spectrum of MoO_x_; the peaks shape shows only one chemical state that can be attributed to (Mo^VI^) species^154^, in the other hand, (Mo^IV^) species has not detected^155^. Hence, we suppose that Mo_8_O_23_ and MoO_2_ are deposited on the surface of ZnO in which the upper layer is rich with Mo_8_O_23_. The idea of two separate layers over each other is not supposed here as both of the two molybdenum oxides are detected by XRD at early stage of reaction (Fig. 19). Figure 21 shows the O 1 s XPS spectrum of ZnO. The binding energy peaks located at 531.1 eV are attributed to oxygen ions^155^. Meanwhile, Fig. 22 shows the Zn 2p double spectra of ZnO. The binding energies of Zn 2p_1/2_ and 2p_3/2_ for Zn^2+^ correspond to the peaks at 1044.7 and (1021.7–1021.8) eV, respectively^156^. Moreover, the calculation of the modified Auger parameter leads to value in agreement with ZnO. The oxygen quantification is also consistent with ZnO attribution. Moreover (~50% O) is only explainable with the presence of ZnO not Zn°. XPS survey on the whole elements is shown in (Supplementary - Figure S11).

The reproducibility of the experiment was checked many times giving the same results. Uncomplete morphology transition of ZnO nanorods and the formation of different ZnO nanostructures decorating the nanoplatelets may be attributed to either the lack of adsorption of PMA anions on some pure ZnO nanorods that caused only formation of flowers and cages like structures. This suggestion is in contradictory with the fact that all reactants are sonicating in homogeneous solution. Other rational reason may be the low concentration of PMA used in the reaction and the need of more reaction time. More experiments using higher concentration of PMA and more reaction time led to the formation of phosphorous and phosphorus oxide.

## Theory for Morphology Transition Engineering (TMTE)

**Figure 23:**
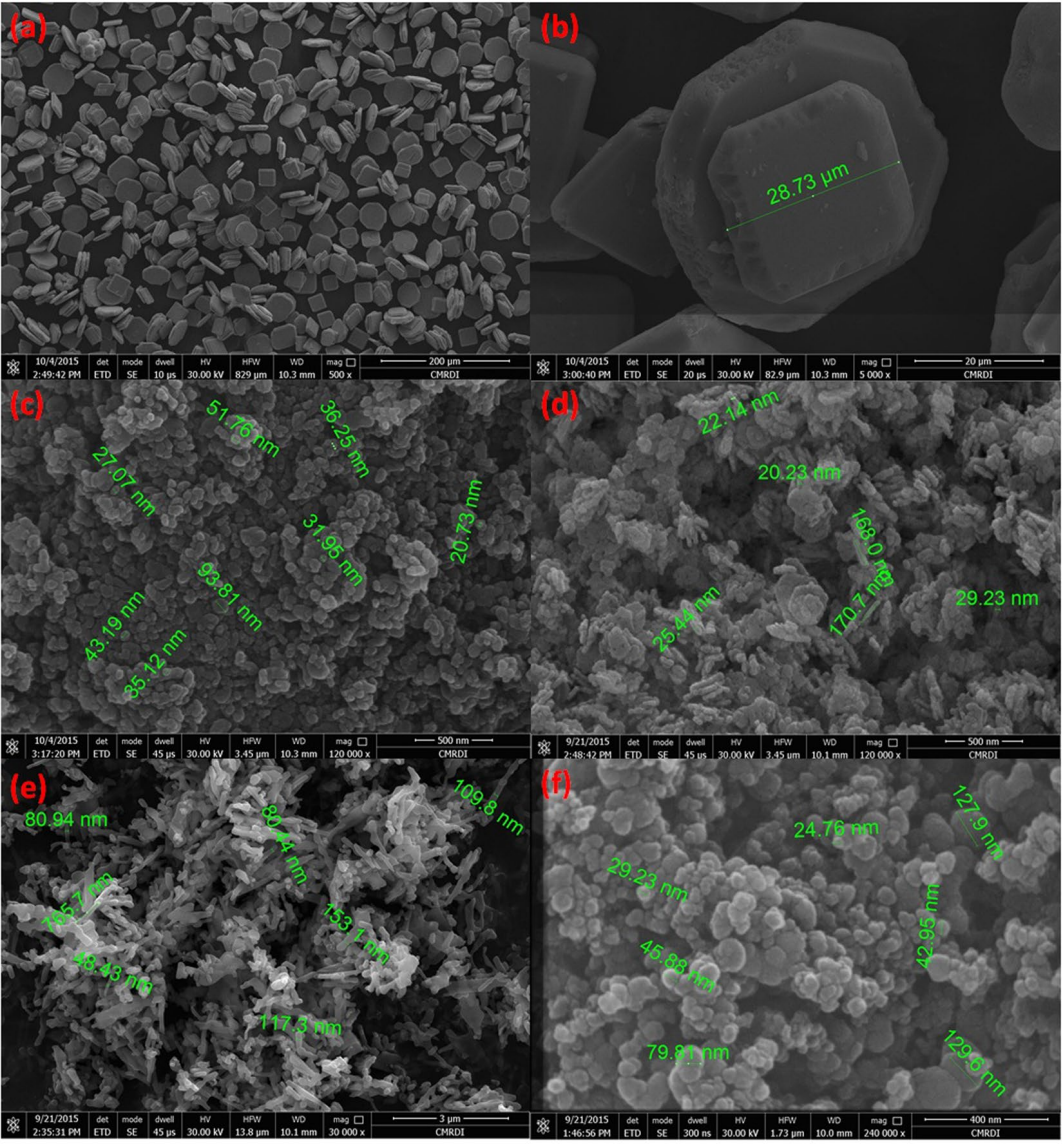
FESEM images of SnO nanoparticles after reaction with POM (**a** and **b**), Fe_2_O_3_ nanoparticles after reaction with POM (**c**), CuO nanoparticles after reaction with POM (**d**), Co_3_O_4_ nanorods after reaction with POM (**e**), and cross-linked carbon nanoparticles (**f**).

Amongst transition metal oxides, zinc oxide (ZnO) shows the largest tendency to experience morphology transition when reacts with phosphomolybdic acid (PMA). As mentioned before in details, the surface basicity or acidity^109^ can account well for ZnO morphology transition. This acidity and basicity of metal oxides are dependent on the charge and the radius of the metal ions as well as the character of the metal oxygen bond. This bond between oxygen and the metal is influenced by the coordination of the metal cations and the oxygen anions, besides the filling of d-orbitals which is specific for each metal oxide. It is a fact, transition metal oxides acquire a unique electron configuration with a specific trend in filling the 3d-sub-shell (Sc^2+^: [Ar] 3d^1^ to Zn^2+^: [Ar] 3d^10^)^157^. This trend is responsible for the fluctuation in the surface chemistry of their oxides. We treated other metal oxides nanostructures like (CuO, Fe_2_O_3_, Co_3_O_4_, and SnO) with PMA, and have not experienced any remarkable morphology transition at the same conditions in which ZnO nanorods was engineered to nanoplatelets as shown in Fig. 23 (Supplementary – Figure S12). Due to the fact that both of copper monoxide (Cu_2_O) and zinc dioxide (ZnO) have completely filled 3d-orbital and vacant 4s-orbital^157^, we expected a similarity in their chemistry when reacts with polyoxometalates at the same reaction condition. Furthermore, amphoteric silver monoxide (Ag^+1^: 4d^10^) and gold monoxide (Au^+1^: 5d^10^) may or may not obey our theory and experience MT. If there are any possibility to apply this theory for binary compounds, cadmium and mercury compounds with closed d-shell may response to POMs and experience MT.

**Figure 24:**
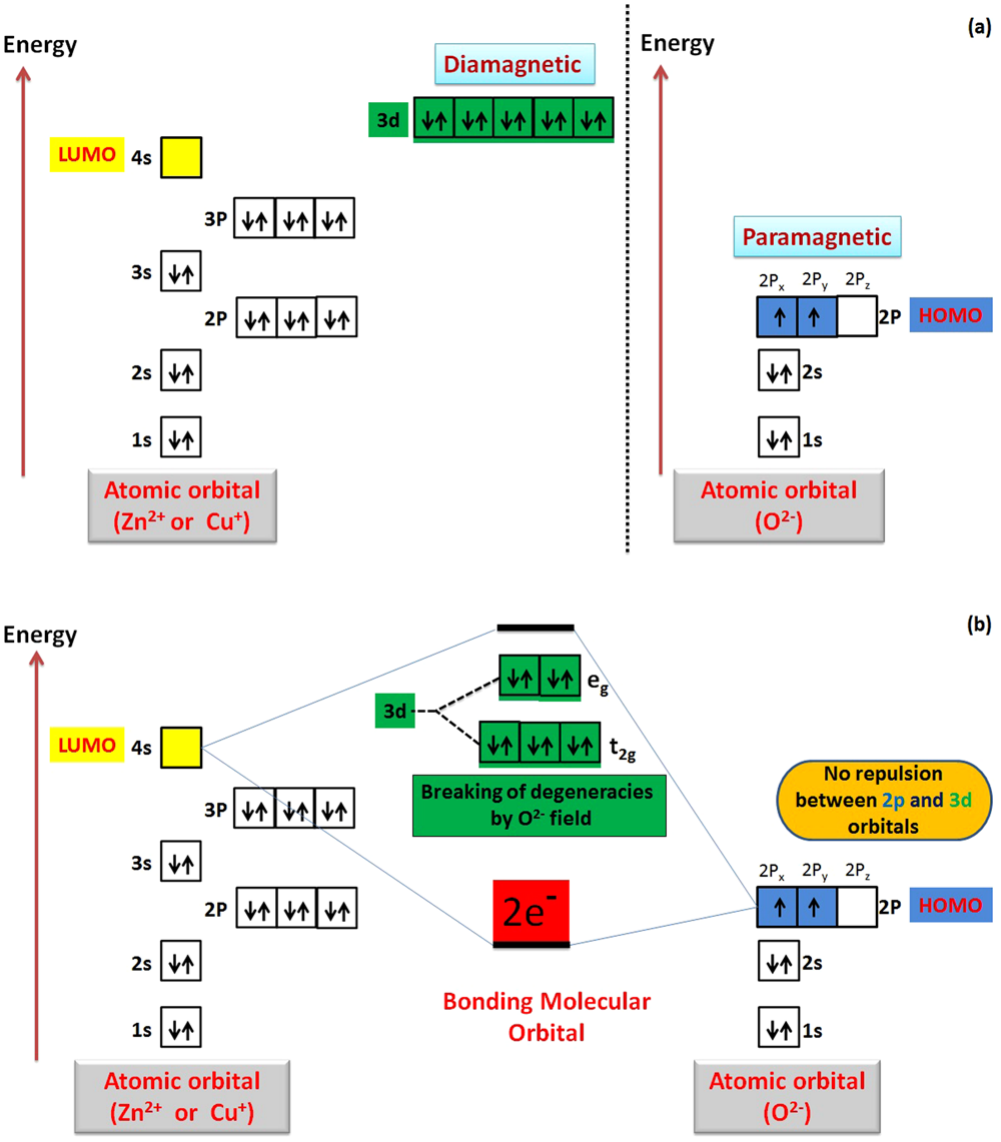
Schematic illustration for splitting of 3d-orbital of cation in the static field of ligand (oxygen anion) and atomic orbitals interaction.

In other words, magnetic properties of cations that form the binary compounds may account well for the fluctuation of their behavior when react with POMs^158–160^. The anions (e.g., O^2−^) acquire paramagnetic properties due to the two unpaired electrons in 2p-orbital; as a consequence they have weak positive susceptibility to magnetic fields. Binary compounds of diamagnetic cations like (3d^10^: Zn^2+^ and Cu^+^) that have a weak negative susceptibility to magnetic fields have more possibility to follow our theory (ATMTE). In this case, both of d-orbital of cation and p-orbital of anion have opposite magnetic behavior, and there is no repulsion between them. So, the latter can bind easily to the 4s-orbitals of cation to form a stable molecular orbital as shown in Fig. 24. Other diamagnetic cations like (Sc^3+^ and Ti^4+^) may follow the same pattern.

In contrast, oxides of cations like (V^+^, Cr^2+^, Mn^2+^, Fe^3+^, Co^2+^,… etc.) that have unpaired electrons will have a fluctuation in their paramagnetic properties according to the number of unpaired electrons in d-orbital. As the Para-magnetism of the cation increases, the repulsion between its d-orbital and 2p-orbital of anion increases which prevent the binding of the latter with 4s-orbital of cations, and prevent the morphology transition process. This may account for the different response of metal oxides to POMs. Hence, *we* state the third selection rule (SR_3_); as follow “*The completely filled 3d-orbital (diamagnetism) of ZnO can account well for its ability to experience morphology transition when react with POMs and recommend other binary compounds of cations like* (*Cd*^*2*+^, *Hg*^*2*+^, *Cu*^+^, *Au*^+^, *Ag*^+^) *to obey ATMTE*”. Evidence to this selection rule is the disability of CuO to obey (ATMTE) due to the presence of single electron that adds the paramagnetic character to all 3d-orbitals and repel 2p-orbitals of oxygen anions and prevent its binding with 4s-orbital of copper cations (Supplementary - Figure S13).

This point of view initiates the questions about the ability of crystal field theory (CFT) to help in describing the empty-filled interaction between 4 s and 2p-orbitals of zinc cation and oxygen anion respectively. To clear this point of view, ligand field theory (LFT) describes the bonding, orbital arrangement, and other characteristics of coordination complexes, but it cannot be considered to analysis this interaction between cations and ligands^161, 162^. Our point of view which may be right, wrong or need corrections, explain the bonding between cations orbitals with the ligand orbitals on the basis of crystal field theory (CFT)^163^. This theory has been used to describe various properties, but it does not attempt to describe bonding. According to this theory, the d-orbital experience a splitting of its energy level under the influence of static field of anions (O^2−^). The weak field of ligand split the d-orbital into 2 levels (e_g_ (up) and t_2g_ (down) the 4s-orbital). We suppose that, the field created by d-orbitals will be distributed around 4s-orbitals. By this way, this distributed field of d-levels is effective for either the attraction with paramagnetic 2p-orbitals (in case of diamagnetic d-levels e.g. Zn^2+^ or Cu^+^) which induce the bonding, or repulsion against paramagnetic 2p-orbitals (in case of paramagnetic d-levels) which obstruct the bonding. We need to do further experiment to check the ability of empty t_2g_ -levels to compete against 4s-orbital as (LUMO) to bind with 2p-orbital of oxygen anion (Supplementary Figure S13). Super-exchange interaction between metal cations and oxygen anions may give some information about this theory^164^. In this case, inducing magnetic properties in metal oxides may account for possibility of doped or substituted binary compounds (metal oxides) to obey our theory (ATMTE)^165^.

The tendency of zinc oxide to experience morphology transition when react with POMs also attributed to its amphoteric behavior^117, 135^. It has a tendency to dissolve in both acidic and basic medium, this guarantees the presence of dissolved cationic and anionic species during all stages of reactions, as the pH value varies remarkably during the reaction from the highly acidic to nearly basic region as mentioned before. These dissolved species are expected to keep fixing the grain boundaries between fused nanorods till producing well-shaped platelets; especially by controlling the deposition of molybdenum oxides that prevent further incorporation of these species into the lattice. Hence, *we* state the fourth selection rule (SR_4_) as follows; “*The amphoteric nature of ZnO accounts well for its ability to experience morphology transition when react with POMs at specific conditions, and recommend other amphoteric oxides to obey ATMTE*”.

**Figure 25:**
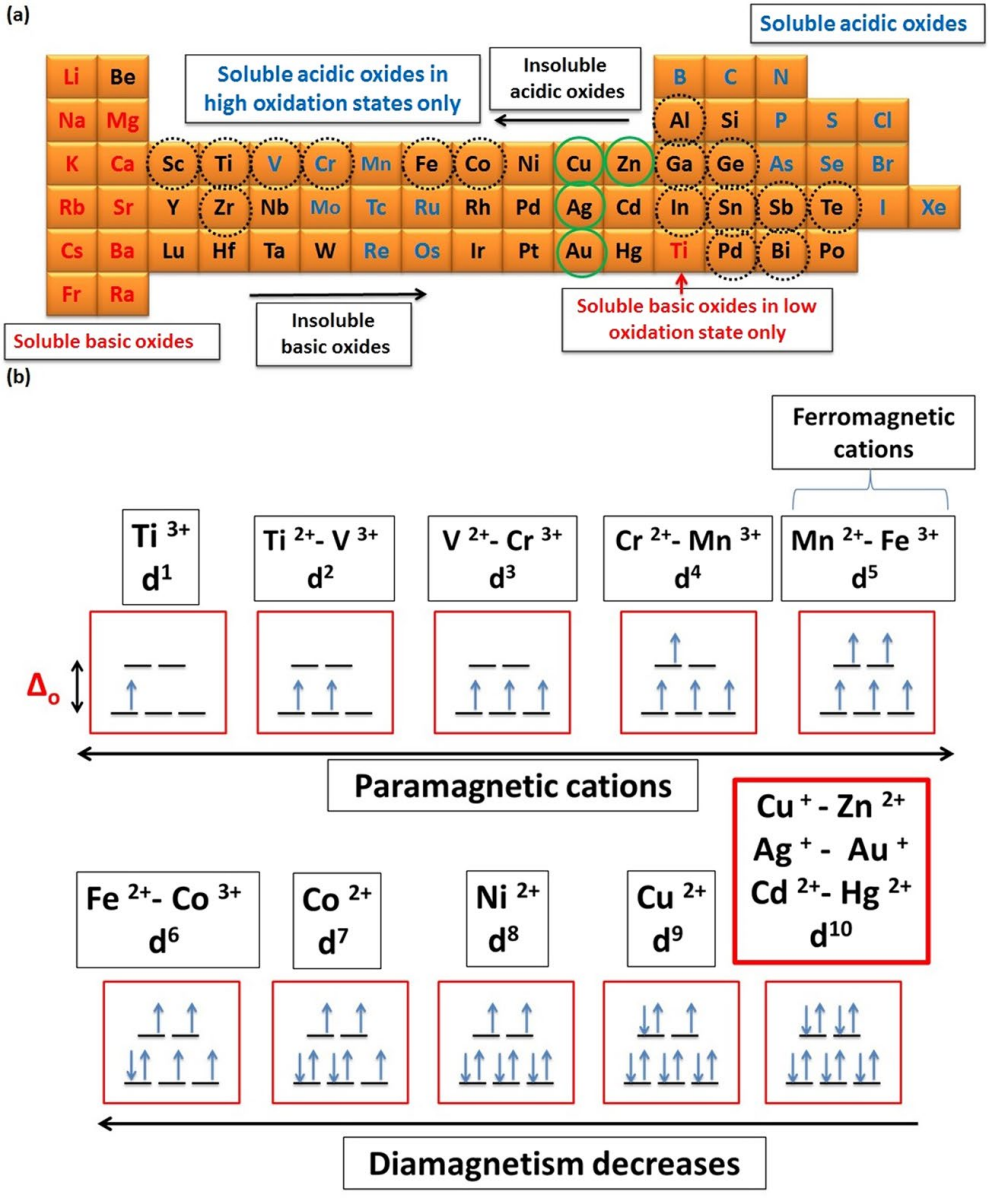
Amphoteric, acidic and basic oxides (doted-circled elements indicate the amphoteric oxides – green circled elements indicate the ideal amphoteric oxides according to ATMTE on the basis of theoretical predictions) (**a**), and magnetic properties of some transition metal cations (**b**).

There are many similarities between the chemistry of elements in group (IB and IIB) (acidic/cationic side) and group (VIA) (basic/anionic side - chalcogens)^164^ which has raised the question about the ability of their binary compounds to experience morphology transition when react with POMs. The elements of group VI [O, S, Se, Te, Po, Lv] with their outer most filled 2p-orbitals are considered good ligands/base in constituting binary compounds with other elements in the periodic table especially group IB and IIB^166^. In other words, a lot of metal oxides obey most selection rules except the rule that states the restriction of 3d^10^ cations (Zn^2+^ and Cu^+^) to obey our theory. Hence, mixing or doping metal oxides with a certain amount of these ideal cations (Zn^2+^) is expected to modify their surface chemistry, and they may have a tendency to experience morphology transition according to our theory (ATMTE). Moreover, doping binary compounds may induce their morphology transition. The importance of this theory is represented in its ability to manipulate synthesizing a variety of hybrid mixed oxides such as [(ZnO grafted MoO_x_ or WO_x_) - (Cu_2_O grafted MoO_x_ or WO_x_) - (ZnO/Cu_2_O grafted MoO_x_ or WO_x_), substituted oxides like [(Zn_x_M_x-n_) oxide, and (Cu_x_ M_x-n_) oxide], and other doped oxides]. In additions, the external morphology of carbonaceous materials decorated with ZnO like (MWCNTs, SWCNTs, all graphene derivatives, CNPs … etc.) will be changed to different dimensions according to reaction conditions as along with deposition of MoOx/WO_x_ on ZnO surface. Miscellaneous nano and micro-structures will be produced either by using different starting structures like (tubes, rods, sphere, particles, sheets, platelets, and hollow structure … etc.) or by varying the reaction conditions. Since, amphoteric behavior is one of the most important properties that should be exhibited by binary compounds (metal oxides) to obey our theory; we recommend oxides of elements that are circled in Fig. 25 to obey our theory^123, 130^. Hence, we expect that these metal oxides may response to POM and experience morphology transition under certain circumstances, for instance by doping or mixing with ideal cations (Zn^2+^ or Cu^+^) that have filled d-orbitals or by increasing the concentration of POMs.

On the basis of all the aforementioned theories, facts, predictions, and observations that were discussed within the whole article, we introduce Theory for Morphology Transition Engineering (TMTE) which states that “Binary compounds especially amphoteric/diamagnetic pure and doped metal-oxides like (ZnO, Cu_2_O) that have appropriate energy difference between their LUMO (acid site/cation) and HOMO (base site/anion), may experience morphology transition to various dimensions (1D, 2D and 3D) when reacts with polyoxometalates under specific conditions, with a possibility to manipulate their surface catalytic properties.

To conclude, we have cut the edge of research in materials engineering and nanotechnology by postulating a series of selection rules constituting a theory for morphology transition engineering (ATMTE). Experimental evidences were discussed by demonstrating the morphology transition of ZnO nanorods to nanoplatelets accompanied with catalytic deposition of Mo_8_O_23_-MoO_2_ mixed oxides. Strong scientific facts that support the possibility to apply this theory to other binary compounds especially metal oxides under certain circumstances were discussed. This theory is expected to be the basis for reliable engineering of hybrid nanostructures with different dimensions and their functionality. A precise explanation of the nanoscale reaction mechanism was demonstrated. Further work is now in progress in *Catholic University of Louvain* (*ICMN*) to suppose a new mechanism (intermediate compound mechanism(ICM)), study the hybrid nanomaterials that may be synthesized by phosphotungestic acid, study the growth mechanism of Mo_8_O_23_-MoO_2_ mixed oxide, postulate theory for morphology engineering of solid compounds (TMESC), beside applying the theory to various binary compounds and evaluate the possibility of engineering other binary and ternary oxide nanostructures using a similar method and their applications in different fields of science.

## Methods

### Preparation of ZnO nanorods

In a typical synthesis of zinc oxide nanorods, 0.3 M aqueous solutions of metal salts (ZnCl_2_, Aldrich 99%) was prepared, then 0.8 M sodium hydroxide was added dropwise under stirring up to complete precipitation of zinc hydroxide. The resulting precipitate was irradiated with household microwave for 15 minutes followed by filtration, washing and drying at 80 °C overnight.

### Preparation of the nanocomposite (ZnO nanoplatelets grafted Mo_8_O_23_-MoO_2_ mixed oxide) decorated ZnO nanostructures

Phosphomolybdic acid solution [60 ml (in dist. water) – (0.01 M) H_3_PMo_12_O_40_] and zinc oxide powder (1 gm) is mixed in 100 ml beaker and ultra-sonicated for 15 minutes using ultrasonic processor model VCX 500 [Power: 500 W - Frequency: 20 kHz]. The temperature reaches nearly 100 °C. Few portion of the product was picked up at different intervals (3.5, 8.5 and 15 minutes) to follow up the reaction mechanism. The samples were centrifuged, washed and dried at 100 °C for 6 hours.

FESEM analysis [CMRDI, Egypt]: Measurements are carried out using an instrument (Model: (FEI- Quanta FEG-250 SEM, Switzerland). A pinch of the powder was sprinkled gently with a spatula on the carbon tape. The stub was tapped to remove the loosely held powder. A blower was then used to remove the extra particles. A drier is further used to make sure of the removal of any other loosed particles and also drying of the moisture content if any.

Size distribution and zeta potential [PSAS, Beni-Suef University, Egypt]: Measurements were carried out using an instrument (Model: Malvern Zetasizer Nano-ZS90). In two beakers (50 ml), few milli-grams of ZnO nanorods and the nanocomposite (decorated ZnO nanoplatelets grafted Mo_8_O_23_-MoO_2_) were dispersed in deionized water using bath sonication. Then, the disposal cuvette “for size distribution measurement” and disposal folded capillary cell “for zeta potential measurement” were filled with few amount of the suspension, and placed in the instrument for investigation.

X-ray diffraction [PSAS, Beni-Suef University, Egypt]: XRD studies were carried out using a diffractometer (Model: PANalytical Empryan 202964-Anode Material: Cu - K_α1_ = 1.54060 °A - K_α2_ = 1.54443 °A - K_β_ = 1.39225 °A - Generator setting at 30 mA, 40 kV). XRD spectra were recorded for the three samples of the nanocomposite that picked up at three different times [3.5, 8.5, and 15 minutes] during the reaction.

HRTEM analysis [Institute of Physics, Augsburg University, Germany]: Measurements were carried out using an instrument (Model: JEOL JEM-2100F). The samples were dispersed in deionized water using bath sonication then a drop of solution was depositing on carbon-coated copper grids and allowing solvent evaporation at room temperature. We have selected platelets with different dimensions to study the proposed mechanism. Energy-dispersive X-ray spectroscopy (EDX) analysis was used to confirm the elemental composition of various crystals through the samples.

XPS analysis [Université catholique de Louvain, Belgium]: The analyses were carried out with a SSX 100/206 photoelectron spectrometer from Surface Science Instruments (USA) equipped with a monochromatized micro focused Al X-ray source (powered at 20 mA and 10 kV). The samples powder pressed in small stainless steel troughs of 4 mm diameter were placed on an aluminium conductive carousel. The pressure in the analysis chamber was around 10^−6^ Pa. The angle between the surface normal and the axis of the analyser lens was 55°. The analyzed area was approximately 1.4 mm^2^ and the pass energy was set at 150 eV. In these conditions, the full width measured at half maximum (FWHM) of the Au 4f_7/2_ peak for a clean gold standard sample was about 1.6 eV. A flood gun set à 8 eV and a Ni grid placed 3 mm above the sample surface were used for charge stabilization. The following sequence of spectra was recorded: survey spectrum, C 1 s, O 1 s, Zn 2p, Mo 3d and C 1 s again to check the stability of charge compensation with time. The C-(C,H) component of the C1s peak of carbon has been fixed to 284.8 eV to set the binding energy scale. Data treatment was performed with the CasaXPS program (Casa Software Ltd, UK); some spectra were decomposed with the least squares fitting routine provided by the software with a Gaussian/Lorentzian (85/15) product function and after subtraction of a non-linear baseline. Molar fractions were calculated using peak areas normalised based on acquisition parameters and sensitivity factors provided by the manufacturer.

### Associated Content

Supporting Information Available: [Synthesizing of CNPs, carbon template, and core/shell spheres - conceiving and designing the idea - additional characterization data (Figures S1–S13)].

## Electronic supplementary material

Supporting Information
