# Author Correction: Morphology Transition Engineering of ZnO Nanorods to Nanoplatelets Grafted Mo_8_O_23_-MoO_2_ by Polyoxometalates: Mechanism and Possible Applicability to other Oxides

**DOI:** 10.1038/s41598-019-47714-3

**Published:** 2019-08-06

**Authors:** Ahmed H. Abdelmohsen, Waleed M. A. El Rouby, Nahla Ismail, Ahmed A. Farghali

**Affiliations:** 1 Materials Science and Nanotechnology Department, Faculty of Postgraduate Studies for Advanced Science (PSAS), Beni-Suef University, 62511 Beni-Suef, Egypt; 2 Augsburg University, Institute of Physics, Universitätsstrass 1, 86159 Augsburg, Germany; 3 Institute of Condensed Matter and Nanosciences (IMCN), Bio- and Soft Matter, Université Catholique de Louvain, Louvain la Neuve, B-1348 Belgium; 4 Physical Chemistry Department, Centre of Excellence for Advanced Sciences, Renewable Energy Group, National Research Centre, 12311 Dokki Giza, Egypt

Correction to: *Scientific Reports* 10.1038/s41598-017-05750-x, published online 19 July 2017

The original version of this Article contained an error, where ‘Theory for Morphology Transition Engineering’ was incorrectly given as ‘Abdelmohsen Theory for Morphology Transition Engineering’. As a result, in the Abstract,

“Abdelmohsen theory for morphology transition engineering (ATMTE) will be demonstrated through the article, besides a brief discussion about possibility of other oxides to obey this theory.”

now reads:

“Theory for morphology transition engineering (TMTE) will be demonstrated through the article, besides a brief discussion about possibility of other oxides to obey this theory.”

The section heading,

“Abdelmohsen Theory for Morphology Transition Engineering (ATMTE)”

now reads:

“Theory for Morphology Transition Engineering (TMTE)”

In this section,

“On the basis of all the aforementioned theories, facts, predictions, and observations that were discussed within the whole article, we introduce Abdelmohsen Theory for Morphology Transition Engineering (ATMTE)”

now reads:

“On the basis of all the aforementioned theories, facts, predictions, and observations that were discussed within the whole article, we introduce Theory for Morphology Transition Engineering (TMTE)”

Finally, in the same section,

“Further work is now in progress in *Catholic University of Louvain* (*ICMN*) to suppose a new mechanism (intermediate compound mechanism(ICM)), study the hybrid nanomaterials that may be synthesized by phosphotungestic acid, study the growth mechanism of Mo_8_O_23_-MoO_2_ mixed oxide, postulate Abdelmohsen theory for morphology engineering of solid compounds (ATMESC)”

now reads:

“Further work is now in progress in *Catholic University of Louvain* (*ICMN*) to suppose a new mechanism (intermediate compound mechanism(ICM)), study the hybrid nanomaterials that may be synthesized by phosphotungestic acid, study the growth mechanism of Mo_8_O_23_-MoO_2_ mixed oxide, postulate theory for morphology engineering of solid compounds (TMESC)”

