# Supplementary material for: Morphology Transition Engineering of ZnO Nanorods to Nanoplatelets Grafted Mo8O23-MoO2 by Polyoxometalates: Mechanism and Possible Applicability to other Oxides
Source: Sci Rep. 2017 Jul 19;7:5946. doi: 10.1038/s41598-017-05750-x (PMC5517553; doi:10.1038/s41598-017-05750-x)
Supplement: Supplementary file 1 — Supporting Information [file 41598_2017_5750_MOESM1_ESM.pdf]

Supporting Information

Morphology Transition Engineering of ZnO Nanorods to Nanoplatelets Grafted Mo_8O_{23}-MoO_2 by Polyoxometalates: Mechanism and Possible Applicability to other Oxides

Ahmed.H.Abdelmohsen,[¥,§,┤,*] Waleed.M.A.El Rouby,[¥,*]Nahla Ismail,[α]Ahmed.A.Farghali[¥]

[¥] Materials Science and Nanotechnology Department, Faculty of Postgraduate Studies for Advanced Science (PSAS), Beni-Suef University, 62511 Beni-Suef, Egypt., [§] Augsburg University, institute of Physics, universitätsstrass 1, 86159 Augsburg, Germany., [┤] Institute of Condensed Matter and Nanosciences (IMCN), Bio- and Soft Matter, Université catholique de Louvain, Louvain la Neuve, B-1348 Belgium., and [α] Physical Chemistry Department, Centre of Excellence for Advanced Sciences, Renewable Energy Group, National Research Centre, 12311 Dokki, Giza, Egypt.

(A) Carbonaceous Polysaccharide as a Template for Fabricating C/SiO_2 Core-Shell Nanostructure:

In a typical procedure, glucose (50 g) was dissolved in deionized water (500 mL) to form a clear solution. The solution was then sealed in a 700 mL autoclave with a Teflon seal and maintained at 170 ^oC for 7 and 8 hours. The autoclave was cooled at air for 20 h until settling of product and suspending of impurities (non-uniformed structures). Black or dark purple puce products were obtained after centrifugation at 5000 rpm for 20 min. A rinsing process involving five cycles of centrifugation/washing/ redispersion was performed with water or ethanol, respectively. The final samples were obtained after oven-drying at 80 ^oC for more than 4 h. Figure S_1 (a and b) show IR charts for carbon template prepared at 170 ^oC for 7 and 8 hours respectively.

Oven-dried carbonaceous microspheres (100 mg) were evenly dispersed in 0.2M solution of $SiCl_4$ with the assistance of ultra-sonication. The ultra-sonication was continued for 60 min to ensure sufficient diffusion of the metal ions into the surface layer. A rinsing process involving 3–5 cycles of centrifugation/washing/redispersion was performed with either water or ethanol, according to the solvent used initially. The black or puce samples obtained were oven-dried at 80 ^oC for 4 h and used as precursors of the hollow spheres. The samples obtained after ion absorption as described above were transferred to alumina crucibles, and these were placed in a muffle furnace. Calcination was carried out in air. In typical procedures, the calcination parameters were set as 500 ^oC for 1 h. The furnace was then left to cool to room temperature. As-formed products were accumulated at the bottom of the crucibles. TEM image and EDX of C/SiO_2 core-shell sphere (C- template at 170 ^oC for 8 hours) are shown in Figure S_1 (c and d) respectively.

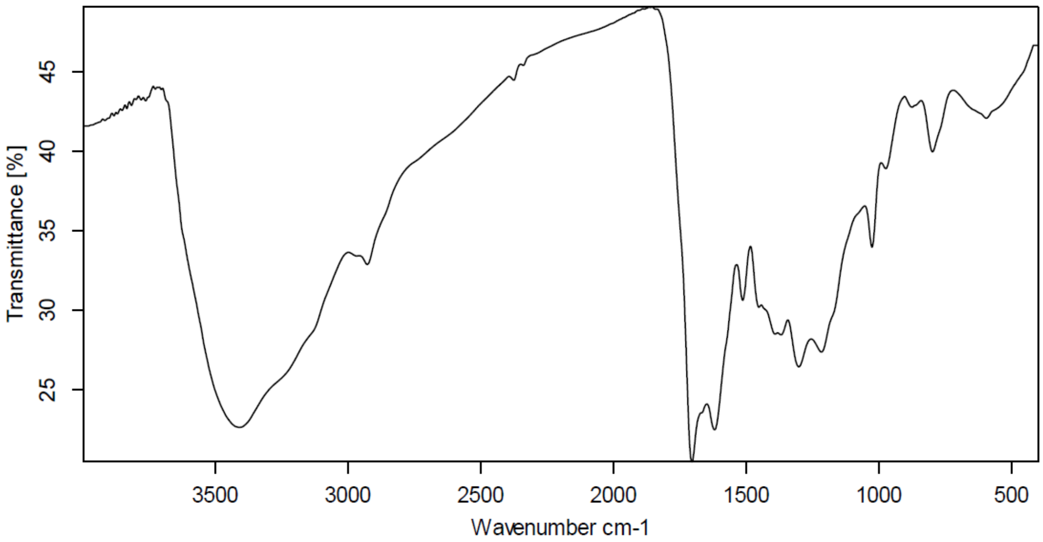

Figure S_1(a): IR chart for carbon template prepared at 170 °C for 7 hours.

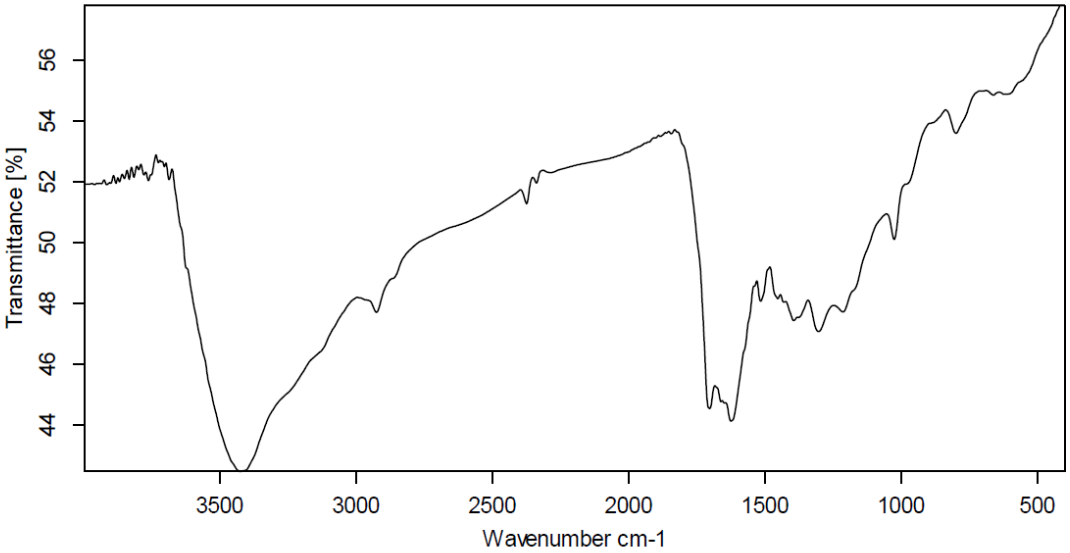

Figure S_1(b): IR chart for carbon template prepared at 170 °C for 8 hours.

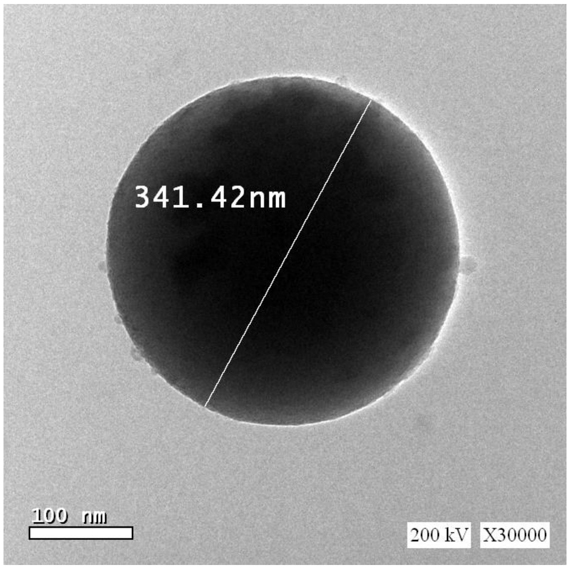

Figure S_1(c): TEM image of C/SiO_2 core-shell sphere (C template at at 170 ^{o}C for 8 hours).

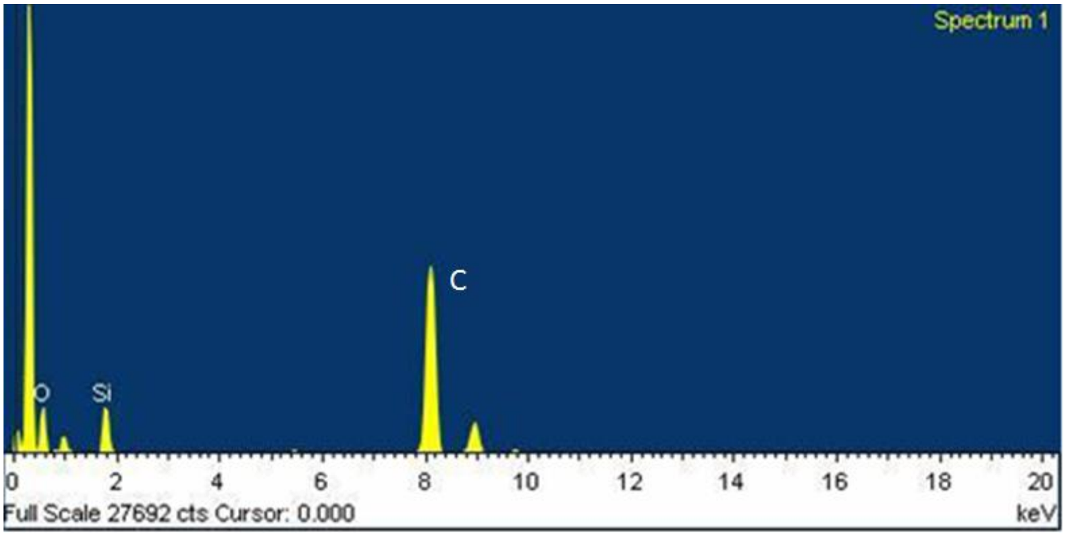

Figure S_1(d): EDX of C/SiO_2 core-shell sphere (C- template at at 170 ^{o}C for 8 hours).

(B) Top-Down Approach for the Preparation of Colloidal Carbon Nanoparticles [Polyoxometalate - assisted solution technique]:

The top-down approach is based on the spontaneous and strong chemisorption of polyoxometalates (POM) on carbon surfaces. We prepared CNPs as follow: a mixture of 1.5 gm Carbon Black in 60 mL deionized water (0.01 M) phosphomolybdic Acid) was ultra-sonicated for 2 hours in which the mixture was centrifuged and the dispersion medium was renewed every 30 minutes (Ultrasonic Processor Model VCX 500 [Power: 500W - Frequency: 20kHz]). At the end of reaction the carbon nanoparticles were collected and dried at 80 ^{o}C for 4 hours. The final product was characterized without pre-treatment with sulfuric acid that used for removing adsorbed MoO_x

on the surface of CNPs which cause formation of crosslinked structure as shown in Figure S_2(a and b). EDX of cross-linked carbon nanoparticles (CNPs cross-linked by MoO_x) is shown in Figure S_2(b).

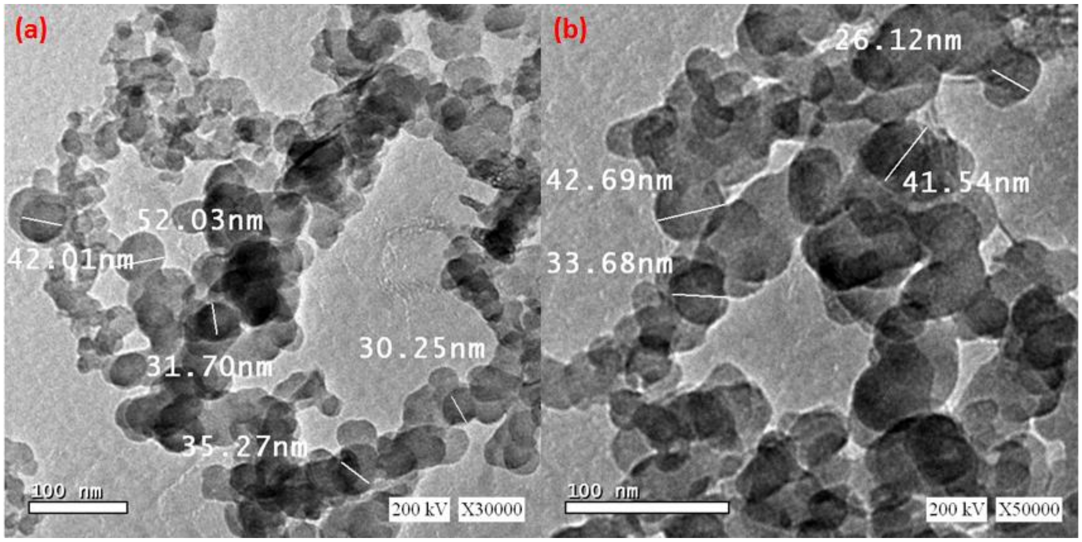

Figure S_2(a): TEM of cross-linked carbon nanoparticles prepared by top-down approach.

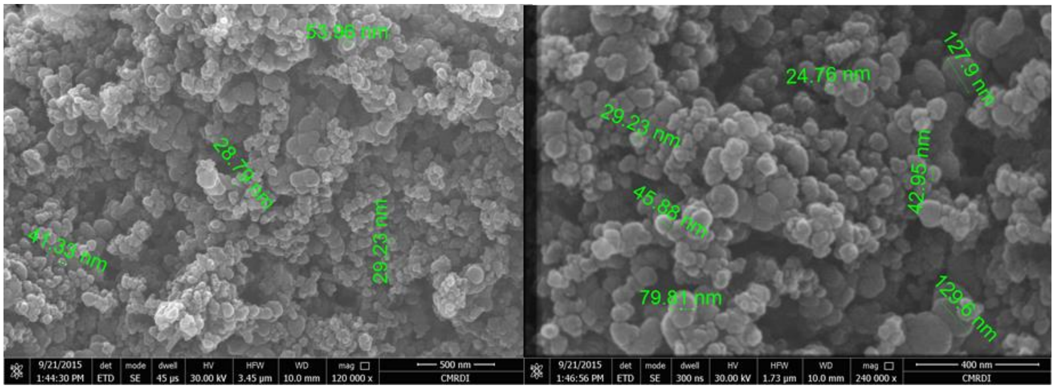

Figure S_2(b): FESEM of cross-linked carbon nanoparticles prepared by top-down approach.

Element	Weight%	Atomic%
O K	70.01	92.24
P K	2.55	1.73
Mo L	27.44	6.03
Totals	100.00	

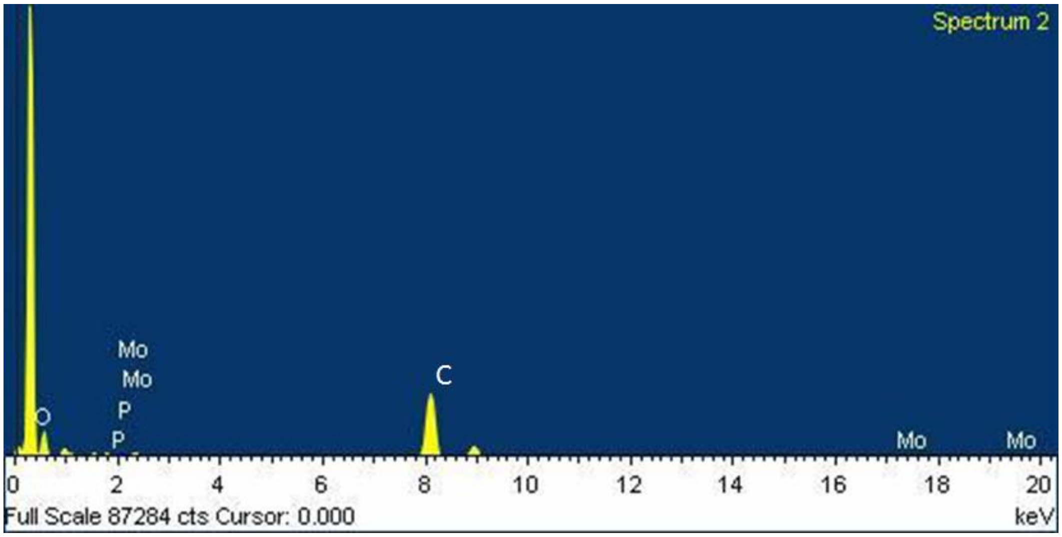

Figure S_2(c): EDX of cross-linked carbon nanoparticles (CNPs cross-linked by MoO_x).

(c) Designing and conceiving the idea [Top-Down Approach for the Preparation of Colloidal Carbon Nanoparticles]:

Abdelmohsen conceived and designed the idea of synthesizing cross-linked metal oxides, by imitating the same approach has been used for prepararion of cross-linked (pre-treated) carbon nanoparticles that were synthesized by top-down approach. Fortunately, due to the difference in chemistry of metal oxides surface in comparing with carbon black, the cross-linked structure experienced further morphology transition to hybride nanoplatelets. We were preparing carbon nanoparticles (CNPs) to be used along with SiO_2@C core-shell sphere as anode material for lithium ion batteries. The CNPs act as the bridging material that prevent agglomeration of SiO2@C core-shell spheres, connect them to the current collector, suppress the volume expansion of silicon oxide (~400%), and enhance diffusion of lithium ions and electrons which enhance the power density of battery. Similarly, the novel idea was conceived and based on designing cross-linked metal oxides to enhance the performance of anodes in lithium ion batteries. Additionally, we may use graphene as an encapsulating conductive agent to afford double protection strategies for the core-shell sphere as along with CNPs. In other words graphene acts as a "buffer zone" for volume variation of SiO_2@C

core-shell spheres and take part in the whole capacity of batery. The expected structure when various metal oxide nanostructures react with PMA is shown in Figure S_3.

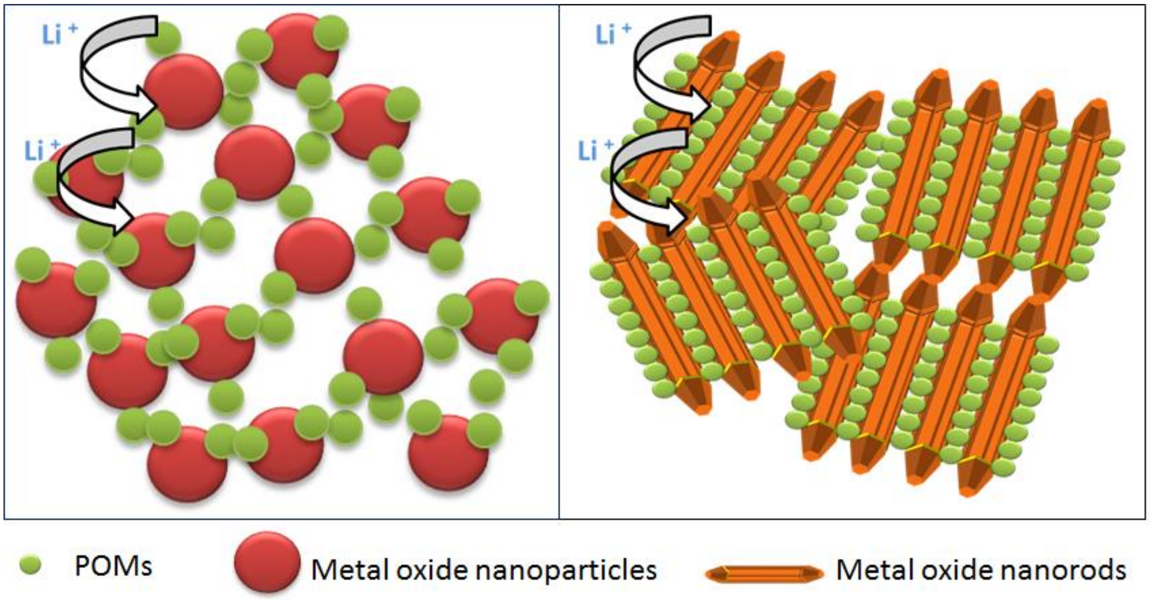

Figure S_3: Expected structure when metal oxide nanoparticles (left) or nanorods (right) react with PMA to be used as electrodes in lithium ion batteries.

(d)Detailed Characterization:

1. Field Emission Scanning Electron Microscope (FESEM) Analysis:

SEM images represent statistical distribution of the composite components (ZnO grafted MoO_x nanoplatelets and decorated nanorods) within the whole sample. As shown in Figure S_4, the ZnO nanorods are well distributed, that is either individually adsorbed on the surface of nanoplatelets or collected closely by few amounts of molybdenum oxides to form flower and cage like structures.

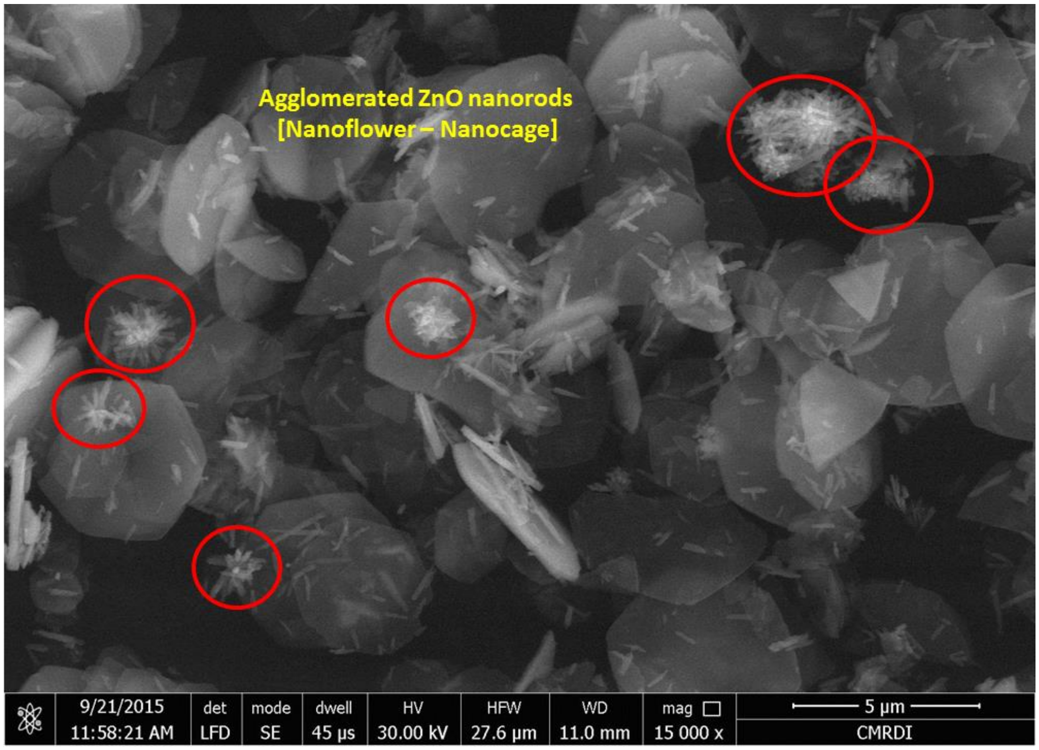
Agglomerated ZnO nanorods
[Nanoflower – Nanocage]
9/21/2015 11:58:21 AM
det LFD
mode SE
dwell 45 µs
HV 30.00 kV
HFW 27.6 µm
WD 11.0 mm
mag 15 000 x
5 µm
CMRDI

٩٩

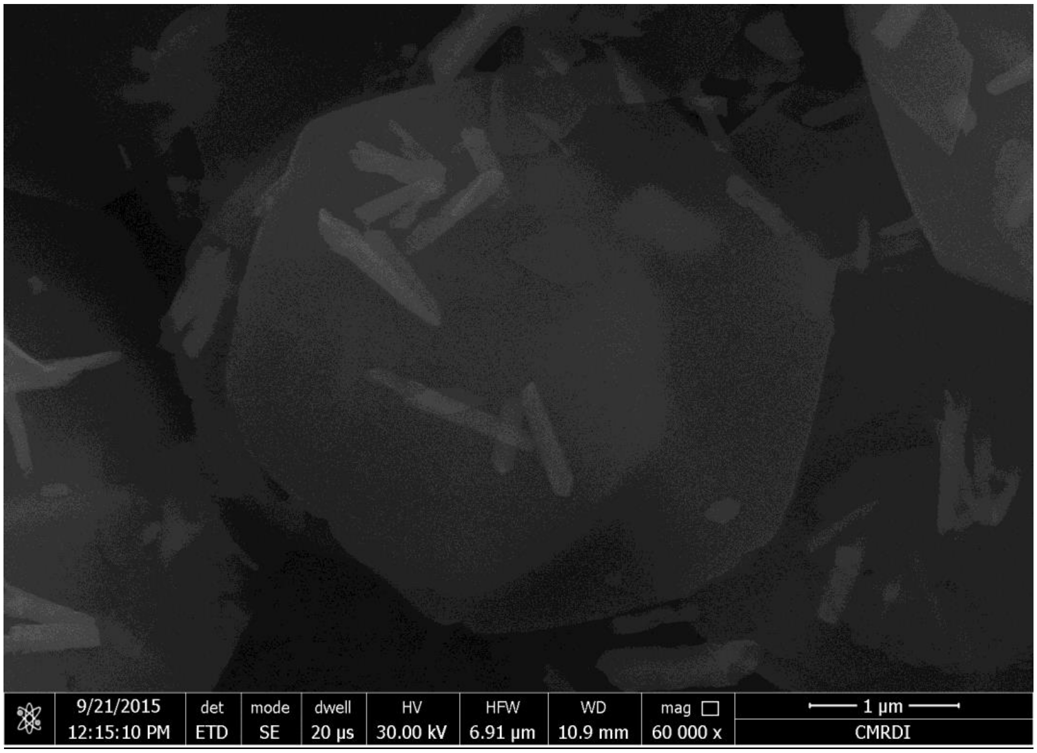
9/21/2015 12:15:10 PM
det ETD
mode SE
dwell 20 µs
HV 30.00 kV
HFW 6.91 µm
WD 10.9 mm
mag 60 000 x
1 µm
CMRDI

١٠٠

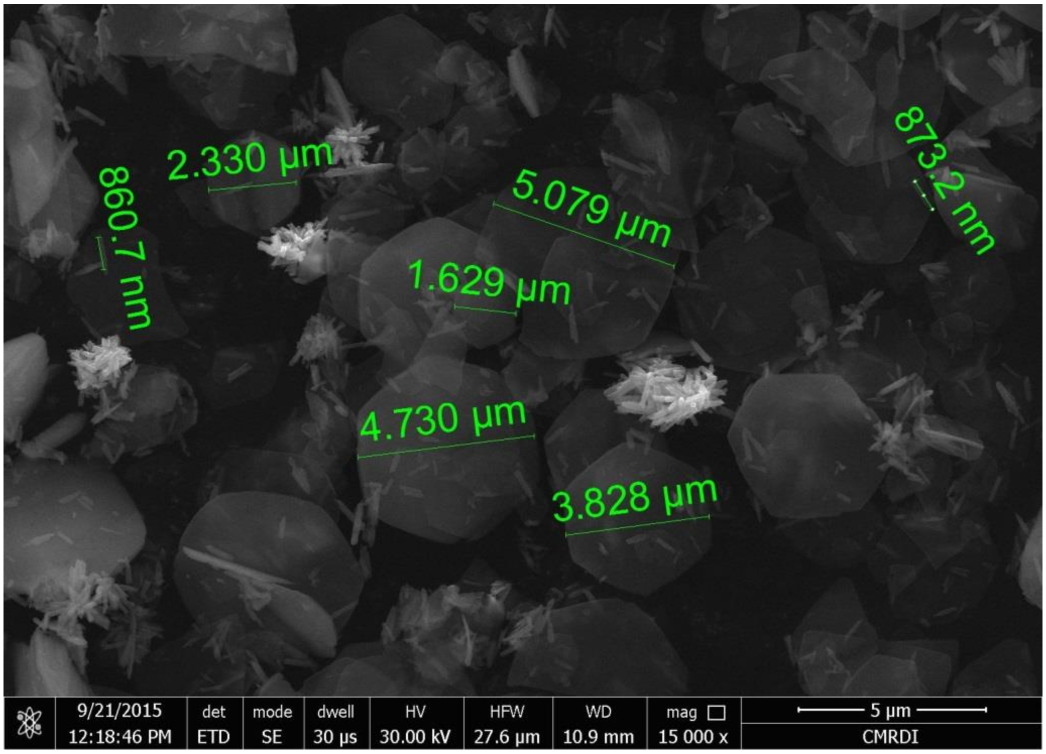

Figure S_4: SEM images of 'decorated ZnO grafted MoO_x nanoplatelets' that illustrate the decorating structures and the dimensions of the nanoplatelets and nanorods over the entire sample (Sample c - 15 minutes).

2. Size Distribution:

Figure S_5 (a,b) shows the size distribution of ZnO and decorated nanoplatelets, respectively. The size distribution' range increased after the morphology transition due to the formation of the nanoplatelets with dimensions larger than that of ZnO nanorods. These values of size distribution well agreed with that determined by FESEM.

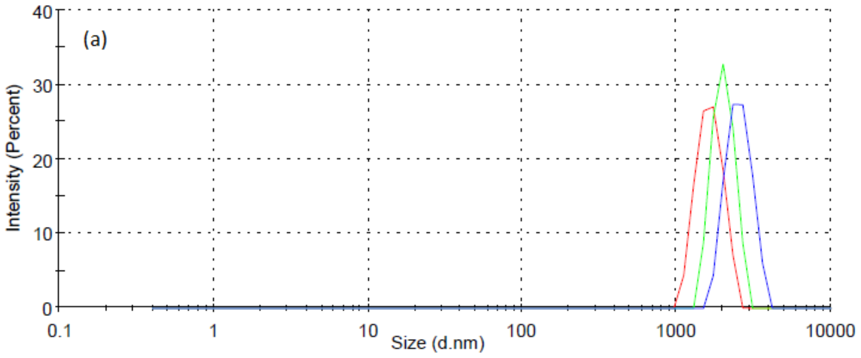

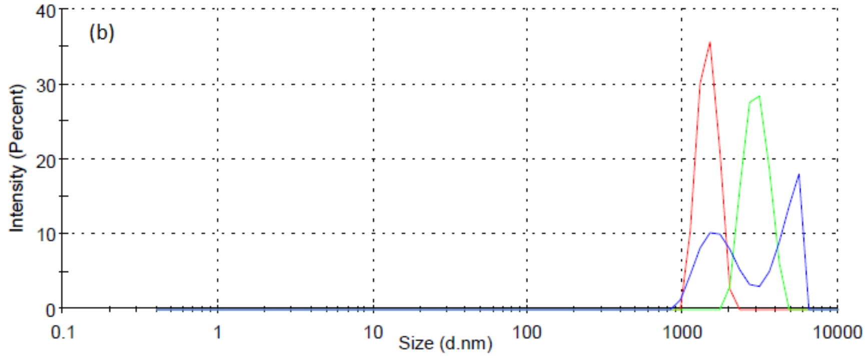

Figure S_5: The size distribution for (a) pure ZnO nanorods and (b) the nanocomposite.

3.Zeta Potential:

Figure S_6 (a,b) show the average zeta potential values for ZnO nanorods and the nanocomposite (23.4 mV and 1.14mV), respectively. The measurements were carried out at pH=7 (lower than IEP of ZnO), so ZnO carried a positive charge, that decreased significantly after immersion in PMA solution which carried a negative charge.

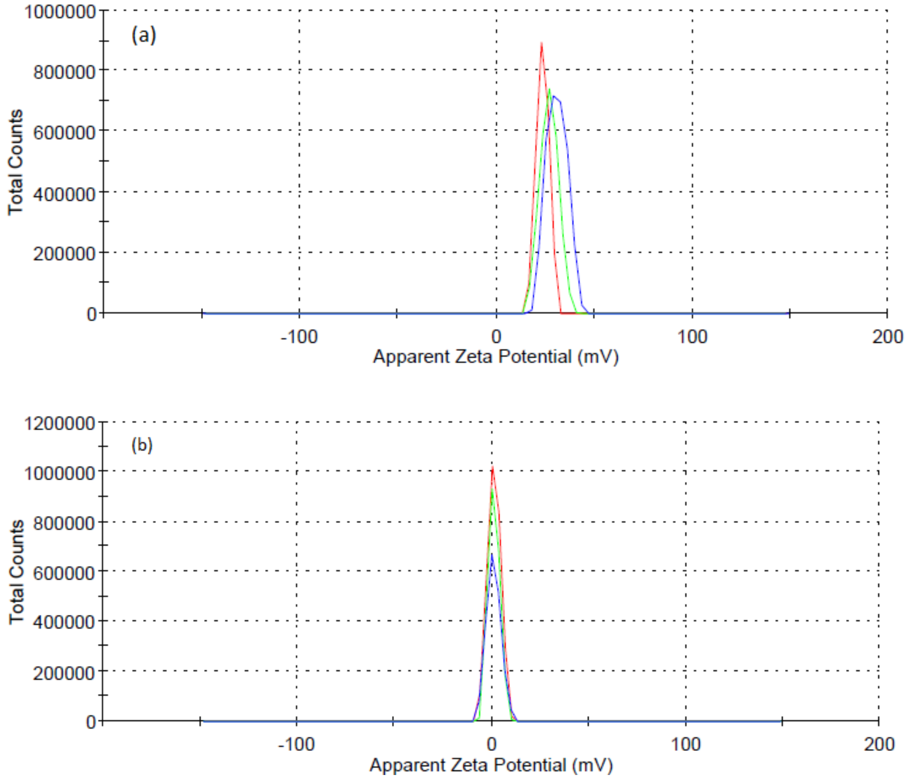

Figure S_6: The zeta potential values for (a) ZnO nanorods and (b) the nanocomposite.

4. Polarization of Ionic Zinc Oxide (ZnO) Bond:

As shown in Figure S_7, cation tends to attract the electron cloud of oxygen anion towards itself because the electron cloud in the anion is loosely held by its nucleus. The anion lose its spherical symmetry and undergoes some distortion. The electron cloud of oxygen ion gets polarised and electron density is pulled in between the nuclei of the two ions. The ionic bond does not remain 100% ionic but develops some covalent character.

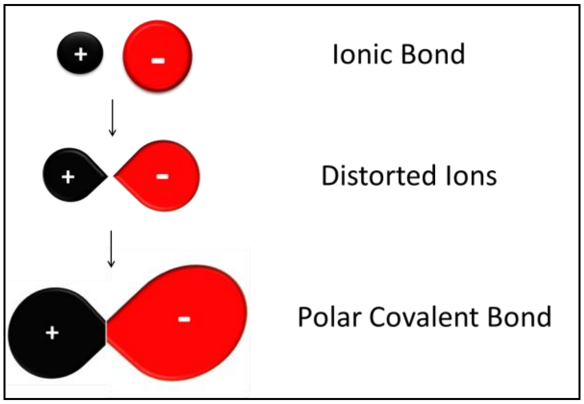

Figure S_7: Development of the polar covalent bond between Zinc cation and oxygen anion.

5. Reaction Progress:

Figure S_8 shows the reaction progresses until complete of reaction.The reaction was completely finished and this was indicated visually, as the characteristic yellow color of POM anions was disappeared completely and the solvent was a transparent. Moreover, the reproducibility of the experiments was checked and revealed the same result.

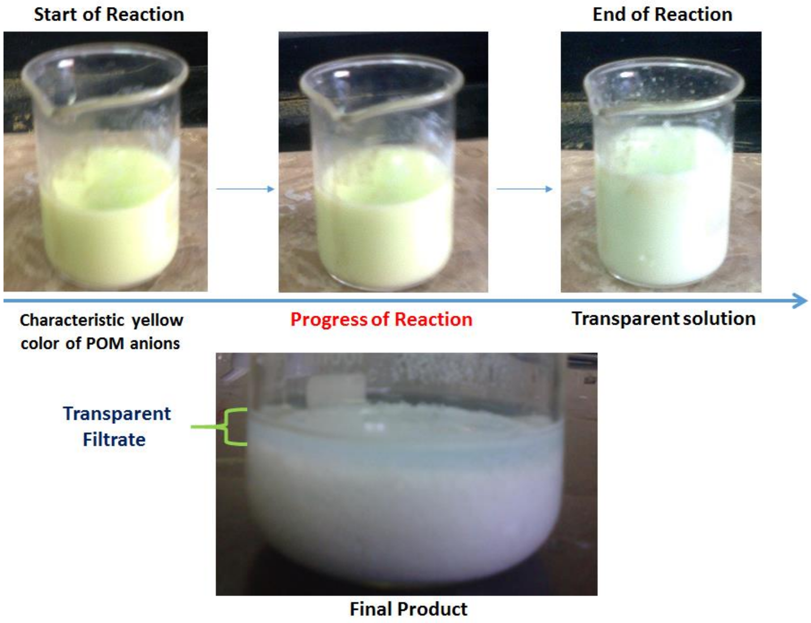

Figure $S_{8:}$ Show the reaction progress to the end of reaction.

6. HRTEM Measurements:

Regarding HRTEM, we have selected platelets with different dimensions to study the proposed mechanism. The contrast of low magnification may interrupt the grain boundary but not justify them. Moreover, the contrast is due to the decorating structures that absorbed on surface which cannot be avoided. This is more obvious in the following HRTEM images (Figure $S_{9(a)}$). HRTEM images show the first step that involves fusion of ZnO nanorods that will be follosed by self-assembly to nanoplatelets are shown in Figure S_9(b).

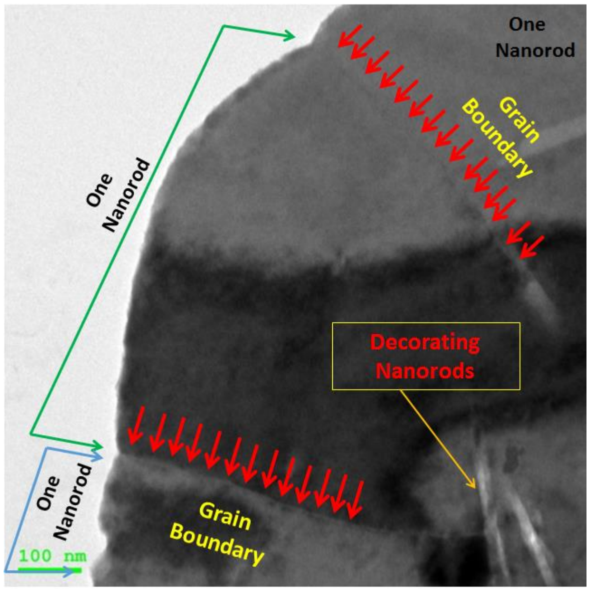

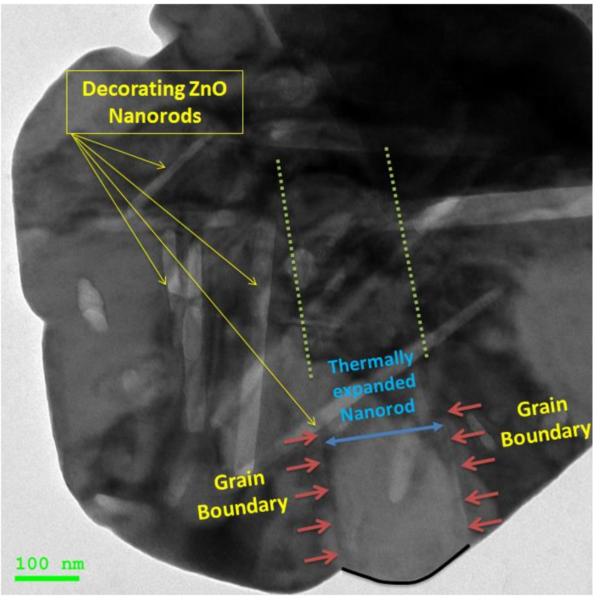

Figure S_9(a): HRTEM for the decorated ZnO grafted MoO_x nanoplatelets illustrate the grain boundaries between fused nanorods [Deposition of MoO_x hinder active sites, and prevent further incorporation of Zn and O species to attain complete fusion].

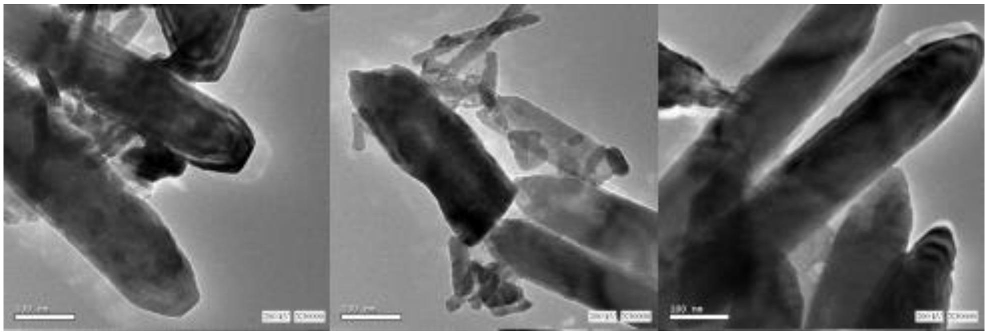

Figure S_9(b): HRTEM images show the first step that involves fusion of ZnO nanorods that will be followed by self-assembly to nanoplatelets.

7.Energy-Dispersive X-ray spectroscopy (EDX):

Energy-dispersive X-ray spectroscopy (EDX) analysis was used to confirm the elemental composition of various crystals through the samples. Oxygen has not taken on account as oxygen of water that was used in sample preparation may affect the accuracy of results. Figure S_{10} illustrates EDX analysis of selected nanoplatelet and ZnO nanorod that decorates the nanoplatelets.

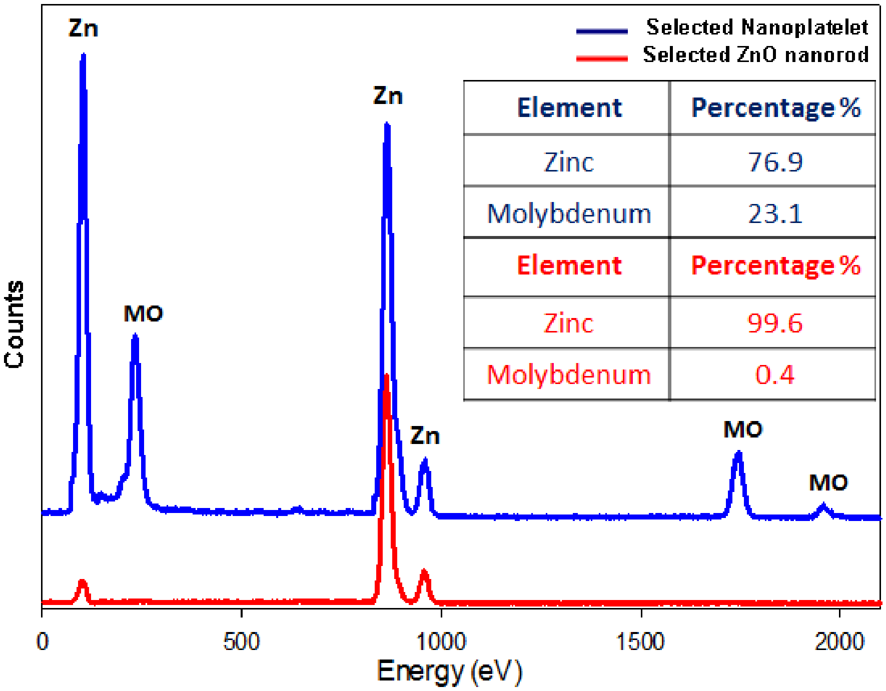

Figure S_{10}: EDX of selected (ZnO nanoplatelets grafted Mo_8O_{23}-MoO_2) and (ZnO nanorod).

8. XPS Analysis:

Figure S_{11} (a and b) show XPS survey for different elements in the energy range (0 – 1100) eV. The adventitious carbon (surface contamination) presents a classical shape of major C-(C,H) contribution and a few oxidized species. The C-(C,H) peak was fixed at 284.8 eV (calibration). Moreover, Cl and P were not recorder with high resolution and the estimated quantification are extracted from survey spectra as shown in Figure S_{11} (a,b). The characteristic XPS spectra for MoO_3 is so similar to that of Mo_8O_{23}, but we recommend the latter as the main peak at $2\Theta = 12.278^{o}$ represents the most characteristic peak of the monoclinic Mo_8O_{23} (011) not of MoO_3. Non-lattice oxygen was dected by XPS analysis, ehich may support also diffusion of oxygen in the outer surface.

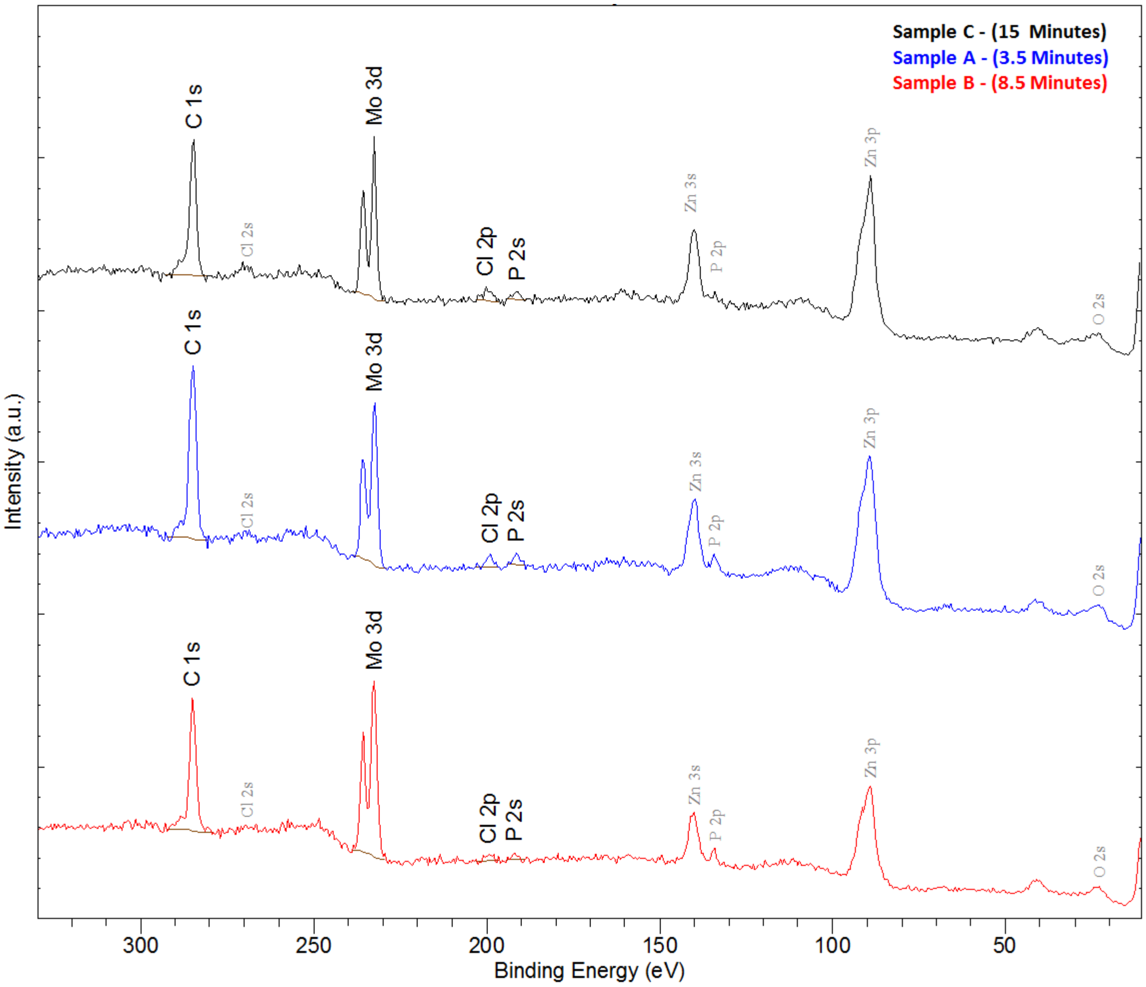

Figure S_{11} (a): XPS survey for three samples of the nanocomposite that were picked up at different intervals (3.5, 8.5, and 15 minutes) after starting of reaction [Energy Range: 0 – 330 eV].

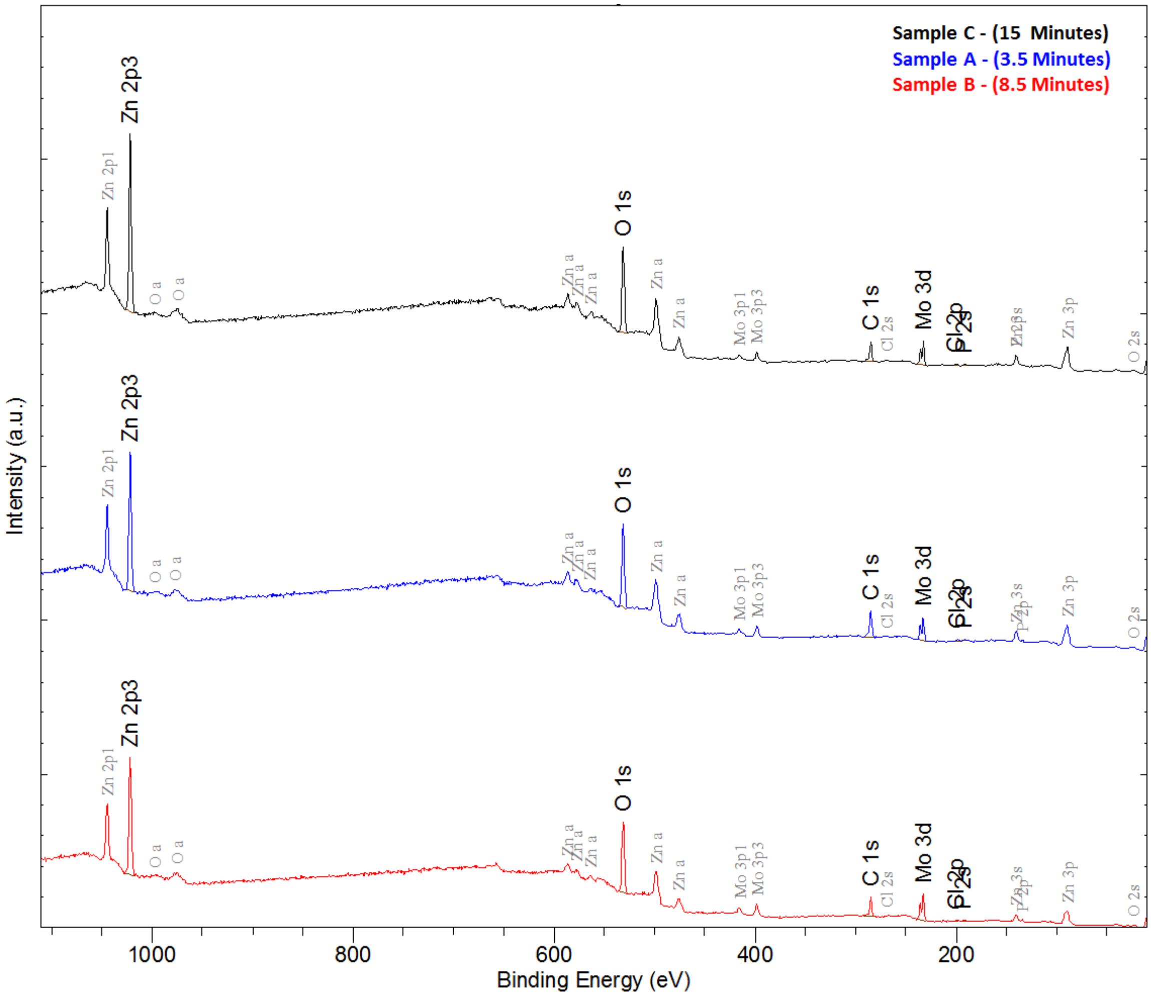

Figure S_{11} (b): XPS survey for three samples of the nanocomposite that were picked up at different intervals (3.5, 8.5, and 15 minutes) after starting of reaction [Energy Range: 330 – 1100 eV].

9.X-ray Diffraction and FESEM:

XRD spectra were recorded for selected transition metal oxides (SnO, Fe_2O_3, Co_3O_4, and CuO) nanomaterials before and after treatment with POMs as shown in Figure S_{12} (a,b,c). All of them were synthesized by microwave assisted solution method. The study reveal that all of the aforementioned metal oxides has not experienced any morphology transition/self-assembly.Hence, This phenomenon seems to be restricted to zinc oxide (ZnO). FESEM images for the metal oxides nanomaterials after reaction with POM are shown in Figure S_{12} (d,e,f).

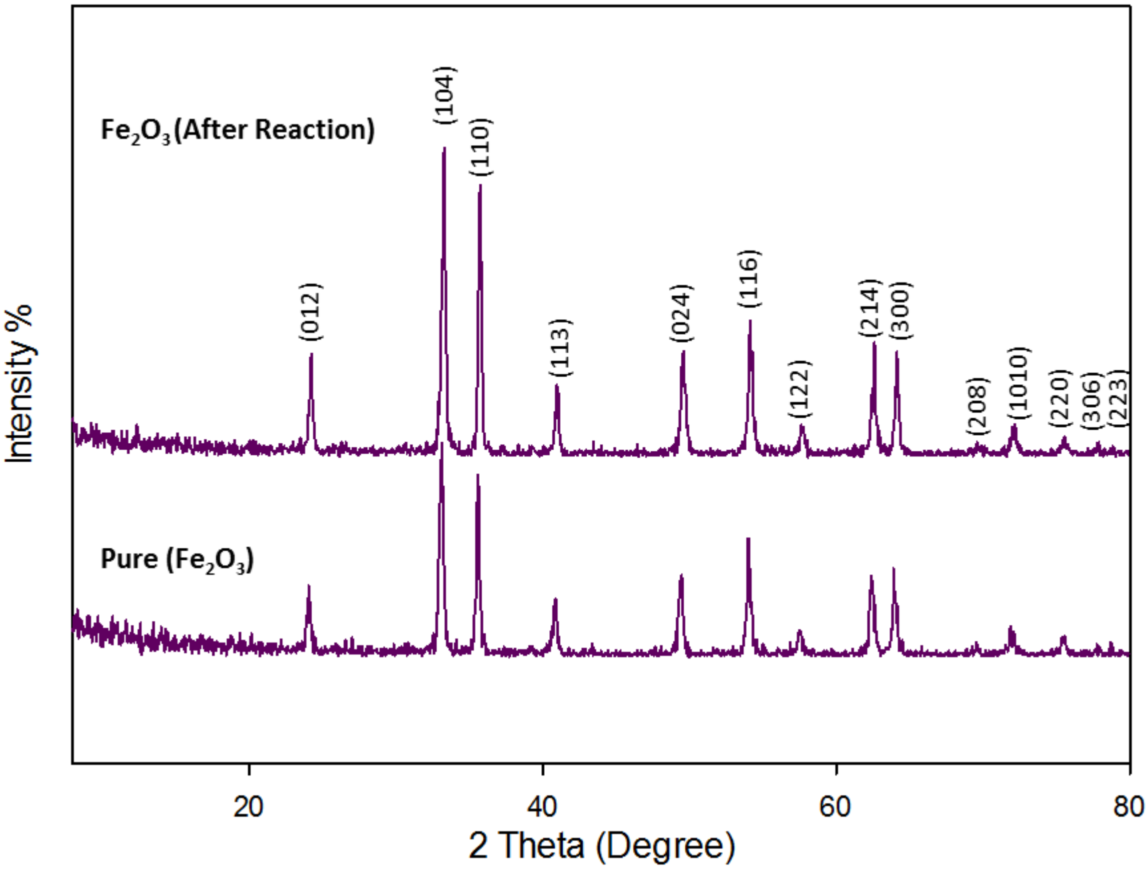

Figure S_{12} (a): XRD Patterns for Pure Fe_2O_3 nanoparticles before and after reaction with POM.

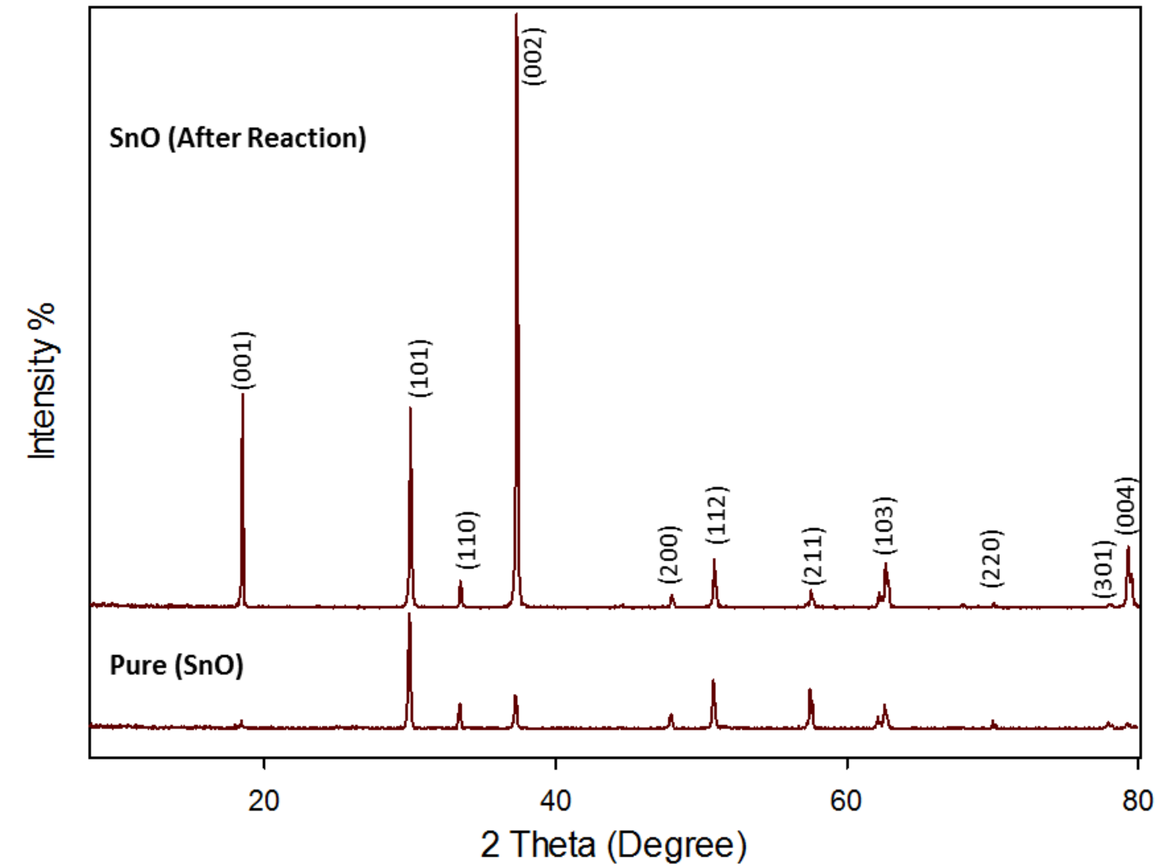

Figure S_{12} (b): XRD Patterns for Pure SnO nanoparticles before and after reaction with POM.

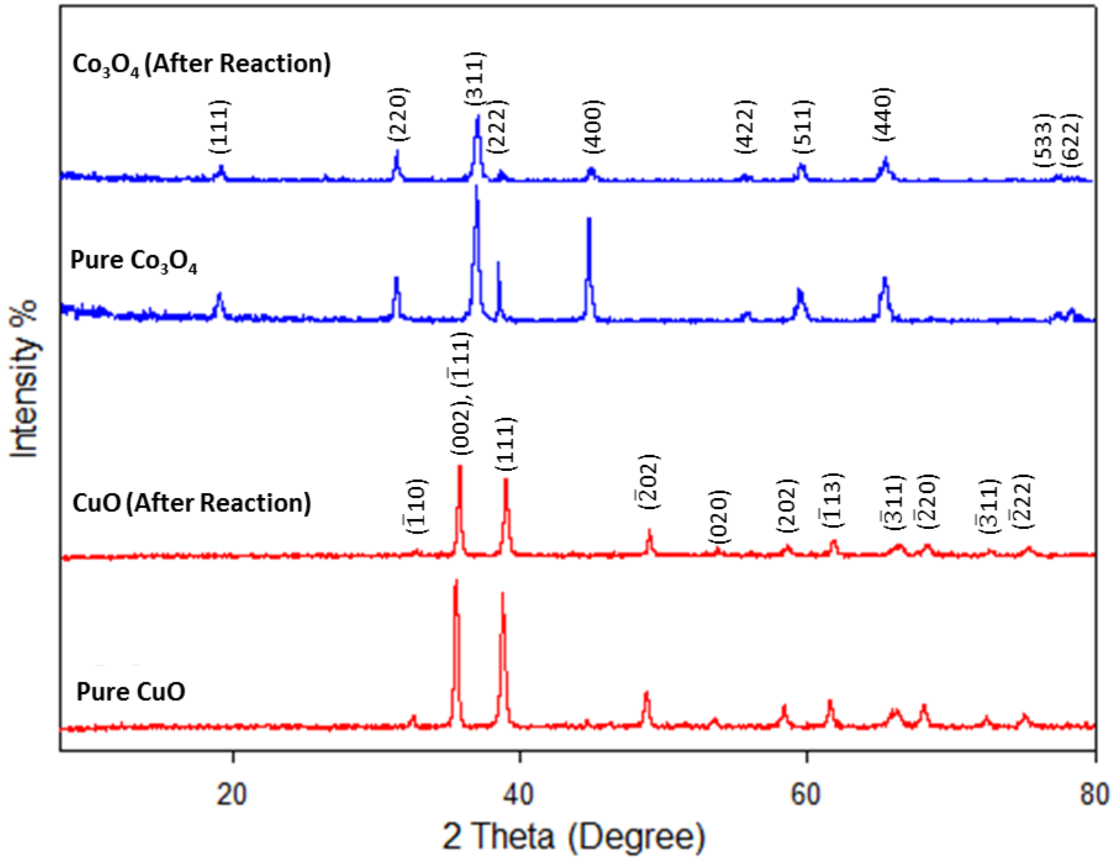

Figure S_{12} (c): XRD Patterns for Pure CuO nanoparticles (down) and Pure Co_3O_4 nanorods before and after reaction with POM.

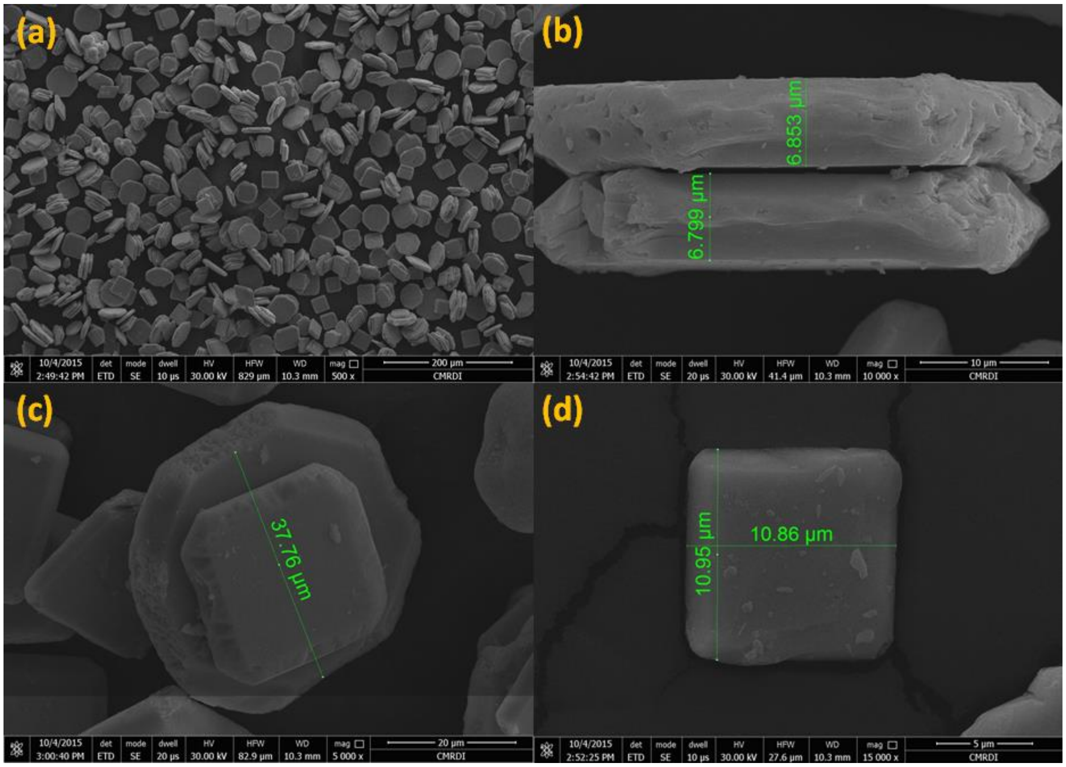

Figure S_{12} (d). FESEM images of SnO nanoparticles after reaction with POM (a,b,c,d).

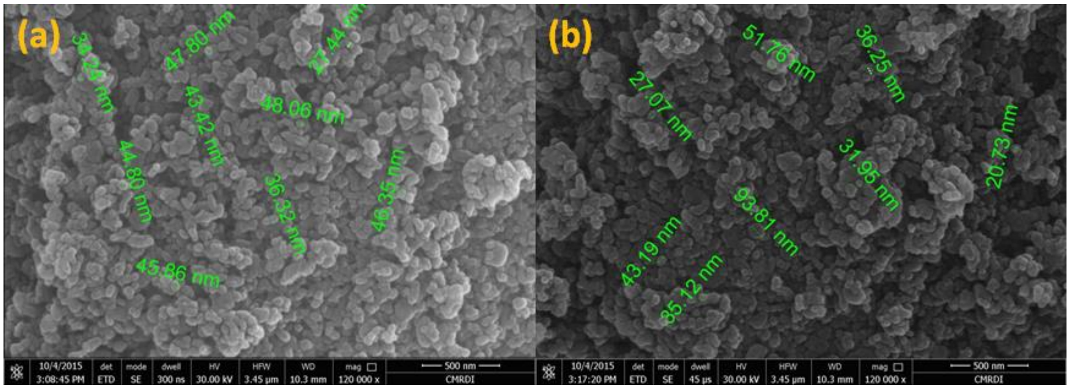

Figure S_{12} (e). FESEM images of Fe_2O_3 nanoparticles after reaction with POM (a,b).

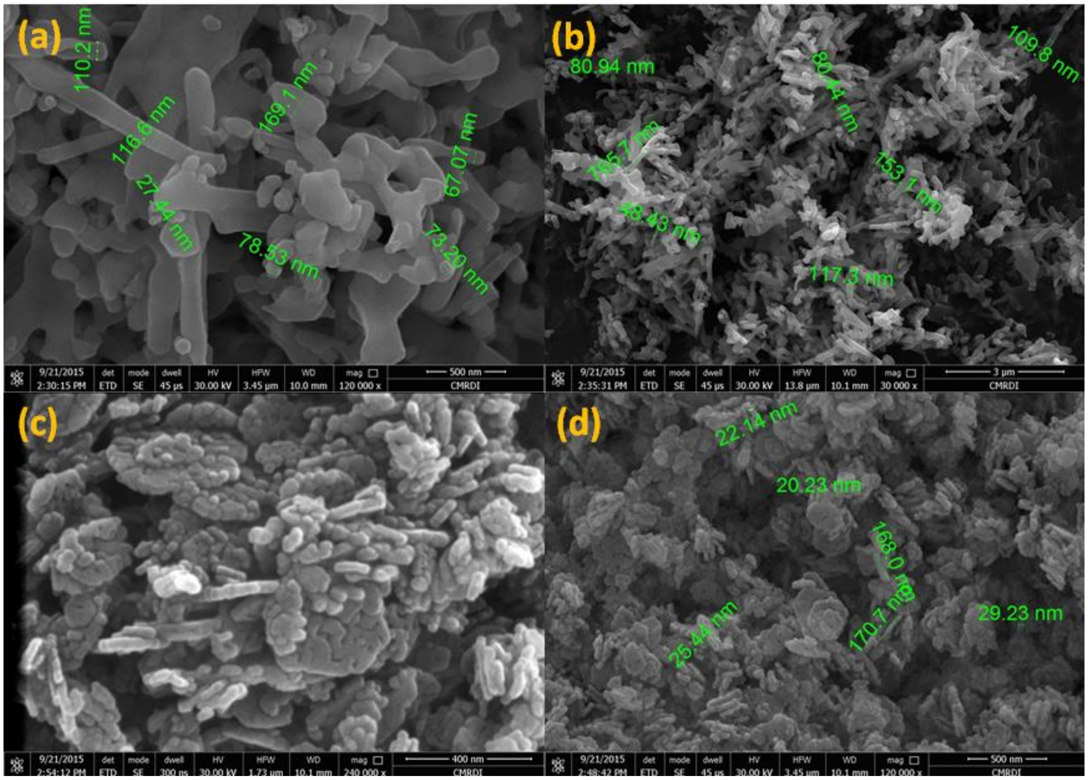

Figure S_{12} (f): FESEM images of Co_3O_4 nanorods (a,b), and CuO nanoparticles after reaction with POM (c,d).

10. Magnetic Properties of Cations and Anions:

Figure S_{13} shows schematic illustration for the ability of crystal field theory (CFT) to help in describing the empty-filled interaction between 4s and 2p-orbitals of zinc cation and oxygen anion respectively, which may account for the possibility of other oxides to experience morphology transition.

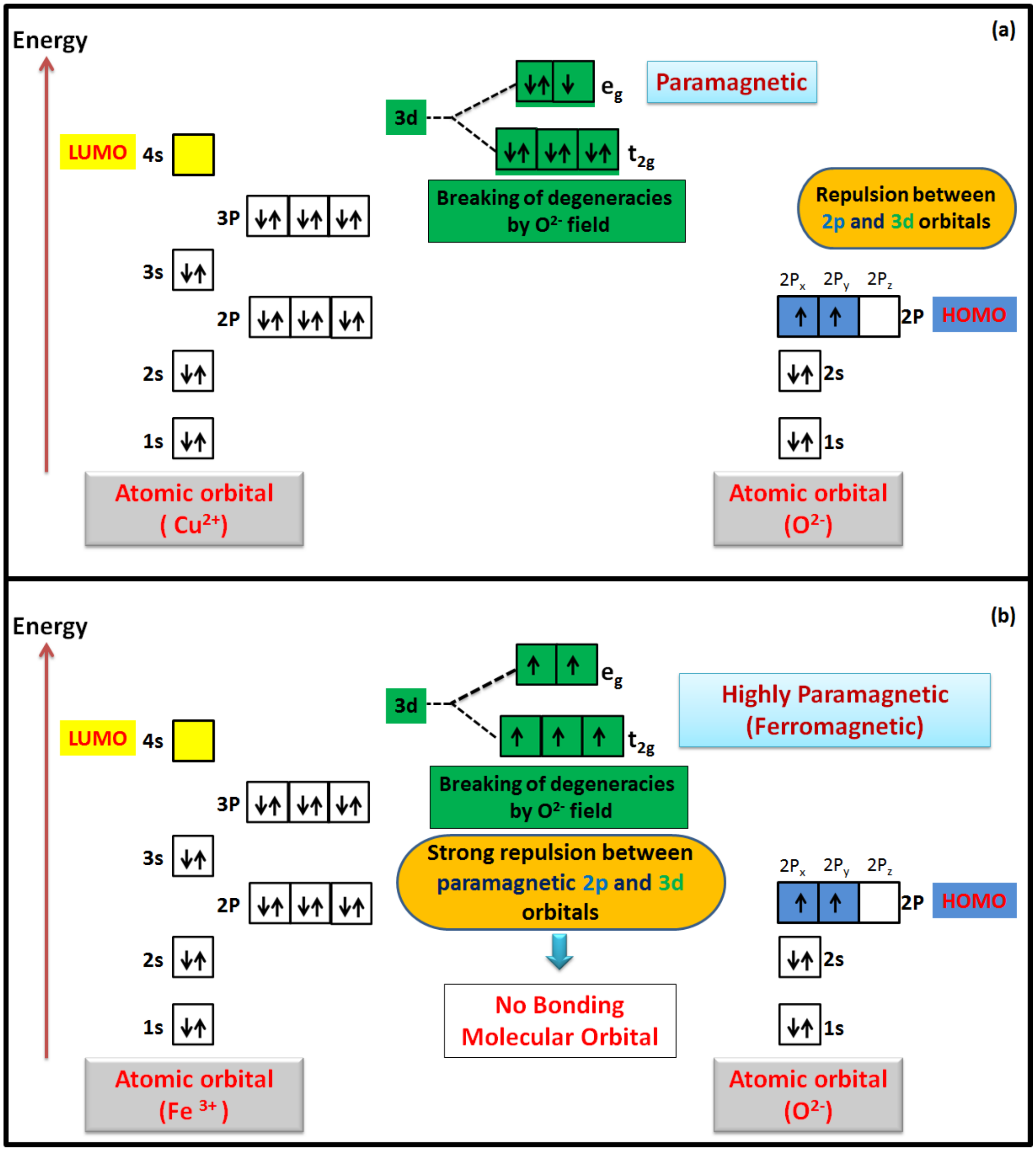

Figure S_{13} (a): Schematic illustration for splitting of 3d-orbital of cation in the static field of ligand (oxygen anion) and obstructing atomic orbitals interaction.

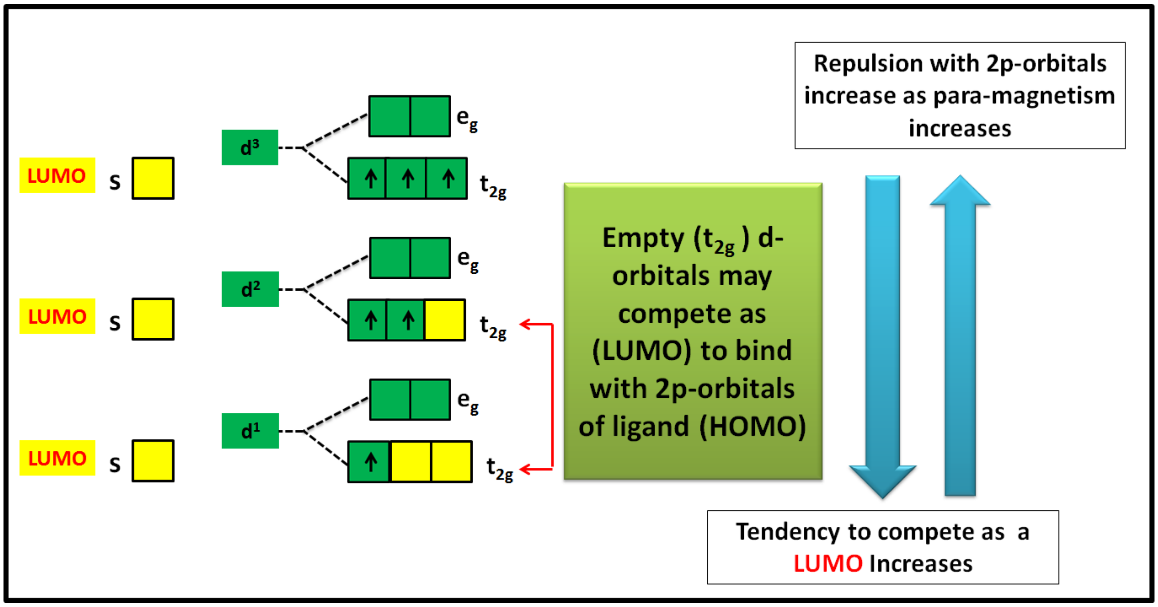

Figure S_{13} (b): Schematic illustration for splitting of 3d-orbital of cation in the static field of ligand (oxygen anion) and expected competeion of d-levels as a LUMO.
